# Akt3 Regulates the Tissue-Specific Response to Copaiba Essential Oil

**DOI:** 10.3390/ijms21082851

**Published:** 2020-04-19

**Authors:** Yasuyo Urasaki, Cody Beaumont, Jeffery N. Talbot, David K. Hill, Thuc T. Le

**Affiliations:** 1 College of Pharmacy, Roseman University of Health Sciences, 10530 Discovery Drive, Las Vegas, NV 89135, USA; yurasaki@roseman.edu (Y.U.); jtalbot@roseman.edu (J.N.T.); 2 dōTERRA International, LLC, 389 South 1300 West, Pleasant Grove, UT 84062, USA; cbeaumont@doterra.com (C.B.); drhill@doterra.com (D.K.H.)

**Keywords:** copaiba essential oil, capillary isoelectric focusing, inflammation, nanofluidic proteomics, protein post-translational modification, neuronal signaling pathways, neuropathic pain, pI3K/Akt/mTOR, JAK/STAT, MAPK

## Abstract

This study reports a relationship between Akt3 expression and tissue-specific regulation of the pI3K/Akt/mTOR signaling pathway by copaiba essential oil. Akt3, a protein kinase B isoform important for the regulation of neuronal development, exhibited differential expression levels in cells of various origins. In neuronal and microglial cells, where Akt3 is present, copaiba essential oil positively regulated the pI3K/Akt/mTOR signaling pathway. In contrast, in liver cells and T lymphocytes, where Akt3 is absent, copaiba essential oil negatively regulated the pI3K/Akt/mTOR signaling pathway. The expression of Akt3 via plasmid DNA in liver cells led to positive regulatory effects by copaiba essential oil on the pI3K/Akt/mTOR signaling pathway. In contrast, inhibition of Akt3 expression in neuronal cells via small interfering RNA molecules targeting Akt3 transcripts abrogated the regulatory effects of copaiba essential oil on the pI3K/Akt/mTOR signaling pathway. Interestingly, Akt3 expression did not impact the regulatory effects of copaiba essential oil on other signaling pathways. For example, copaiba essential oil consistently upregulated the MAPK and JAK/STAT signaling pathways in all evaluated cell types, independent of the Akt3 expression level. Collectively, the data indicated that Akt3 expression was required for the positive regulatory effects of copaiba essential oil, specifically on the pI3K/Akt/mTOR signaling pathway.

## 1. Introduction

For millennia, the oleoresin of *Copaifera* trees has been widely used by indigenous populations of the Neotropics as folk medicine to treat various medical conditions, such as microbial infection, inflammation, and open wounds [1]. In recent years, the demand for copaiba essential oil, the volatile fraction collected via steam distillation of the oleoresin, has steadily increased in the cosmetic, food, and wellness industries [2]. The medicinal properties of copaiba essential oil are supported by the observation that a major component, β-caryophyllene, which constitutes more than 50% of the oil, selectively binds to cannabinoid receptor 2 (CB2) [3]. Modulation of CB2 has been proposed as a viable strategy to alleviate pain and inflammation [4]. Numerous in vitro and in vivo studies support the therapeutic potential of copaiba essential oil to ameliorate inflammatory conditions [5,6,7]. Due to its safety profile, particularly its approval by the U.S. Food and Drug Administration for use as a flavoring agent in food and beverages [8], copaiba essential oil presents a very attractive natural alternative to synthetic pharmacological agents, which are commonly associated with severe side effects. For example, standard treatment of inflammatory arthritis with non-steroidal anti-inflammatory drugs and cyclooxygenase-2 inhibitors is associated with elevated risks of cardiovascular disease and gastrointestinal symptoms [9,10]. However, clinical data on the use of copaiba essential oil for the treatment of inflammatory conditions are lacking. Furthermore, the efficacy of copaiba essential oil for the treatment of any medical condition has yet to be clinically demonstrated in a large-scale randomized controlled trial [11]. 

The medicinal benefits of copaiba essential oil continue to be reported empirically in end users as well as in small-scale clinical studies. Individuals with joint pain and inflammation have reported beneficial effects after using copaiba essential oil. A patient with inflammatory arthritis, who suffered from the side effects of naproxen and ibuprofen during standard care, reported pain relief with no discernible side effects after topical application of copaiba essential oil [11]. In a double-blind, placebo-controlled clinical trial, volunteers with acne vulgaris experienced a significant reduction in the affected surface areas following treatment with copaiba essential oil for 21 days [12]. Participants with rheumatoid arthritis, osteoarthritis, and/or chronic inflammation reported ameliorative effects on hand arthritis following massage-like application of copaiba essential oil with other essential oils [13]. In addition to topical application, other routes of copaiba essential oil administration, such as inhalation in aromatherapy and ingestion of the oil encapsulated in soft gels, are also being used by consumers. The beneficial effects of orally administered copaiba essential oil have been reported in multiple animal studies [14,15,16]. However, clinical studies of the dosages, toxicity, pharmacokinetics, and pharmacodynamics of copaiba essential oil in humans have not been reported [17]. 

Recently, our laboratory revealed that the biological activities of essential oils could be evaluated by measuring their effects on selected signaling pathways in cultured mammalian cells. Signaling pathways are cascades of protein kinases that relay extracellular stimuli to elicit cellular responses. Different signaling pathways are responsive to different stimuli and regulate different cellular processes. We showed that copaiba, mandarin, *Melissa*, and turmeric essential oils could be differentiated by their distinctive effects on selected signaling pathways [18]. In addition, we described distinctive action mechanisms and kinetics of cannabidiol (CBD) oil and copaiba essential oil. Whereas CBD oil had slow and negative regulatory effects, copaiba essential oil had fast and positive regulatory effects on multiple signaling pathways in SH-SY5Y neuronal cells [19,20]. Most surprisingly, we reported that copaiba essential oil had positive regulatory effects on the pI3K/Akt/mTOR signaling pathway in SH-SY5Y neuronal cells, but negative regulatory effects on this pathway in HepG2 liver cells [18,20]. The pI3K/Akt/mTOR signaling pathway regulates cell growth, proliferation, and metabolism [21,22]. The mechanism underlying this tissue-specific response to copaiba essential oil is unclear. However, we observed different compositions of Akt isoforms between SH-SY5Y neuronal cells and HepG2 liver cells. Three Akt isoforms with distinctive functional specificity have been identified in mammals: Akt1, Akt2, and Akt3, which regulate cell proliferation, glucose metabolism, and neuronal development, respectively [23]. SH-SY5Y neuronal cells express all three Akt isoforms [19,20]. In contrast, HepG2 liver cells express only Akt1 and Akt2 [18,20]. We thus proposed that Akt3 was responsible for the differential regulation of the pI3K/Akt/mTOR signaling pathway in liver and brain cells. 

In this study, the relationship between Akt3 and tissue-specific response to copaiba essential oil was further examined. Nanofluidic proteomics technologies were deployed to measure tissue-specific expression levels of Akt3 in various cell types. The effects of copaiba essential oil on selected cell types with differential Akt3 expression levels were evaluated by measuring the phosphorylation states of multiple biomarker proteins within the pI3K/Akt/mTOR signaling pathway. In addition, the expression of Akt3 was modulated in neuronal and liver cells and the impacts of such modulation on the regulatory effects of copaiba essential oil on the pI3K/Akt/mTOR signaling pathway were assessed. Moreover, we examined the relationship between Akt3 and other signaling pathways, such as the MAPK and JAK/STAT pathways, which regulate cell proliferation [24] and immunity [25], respectively. We anticipate that the mechanisms underlying the tissue-specific response to copaiba essential oil could assist in selecting the optimal route of administration.

## 2. Results

### 2.1. Tissue-Specific Expression of Akt3 Isoform

**Figure 1:**
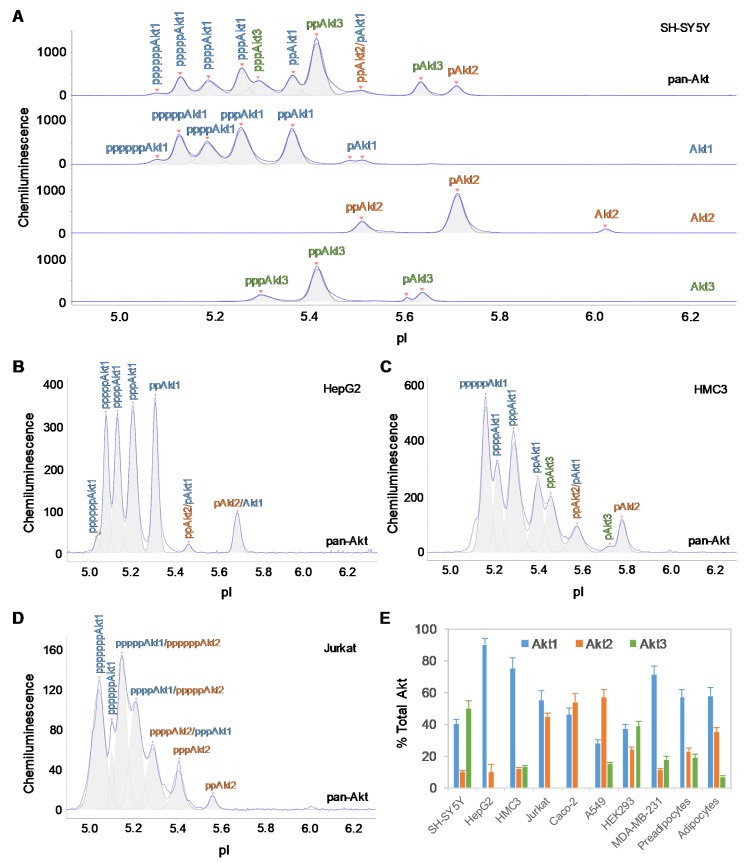
Tissue-specific expression of Akt isoforms. (**A**) Akt isoform identification in SH-SY5Y cell lysates using capillary isoelectric focusing (cIEF) immunoassays. Primary antibodies against pan-Akt (top panel), Akt1 (second panel), Akt2 (third panel), and Akt3 (fourth panel) were used. Identities of Akt isoforms in (**B**) HepG2 cells, (**C**) HMC3 cells, and (**D**) Jurkat cells on cIEF electropherograms. Peaks associated with Akt isoforms are labeled and color-coded: Akt1 (blue), Akt2 (orange), and Akt3 (green). (**E**) Expression levels of Akt isoforms as percentages of total Akt expression level in ten different cell types. The error bars indicate the standard deviations of six repeated measurements per experimental condition.

The tissue-specific composition of Akt isoforms in ten different cell types was examined using capillary isoelectric focusing (cIEF) immunoassays. Primary antibodies that recognize all Akt isoforms (pan-Akt), as well as those specific to the individual Akt1, Akt2, and Akt3 isoforms, were used. In brief, cIEF immunoassays separate proteins by charge in individual nanocapillaries. Following isoelectric focusing, proteins are immobilized by photocrosslinking. Primary antibodies, secondary antibodies, and detection reagents are sequentially introduced. Protein phosphoisoforms separated by charge are detected by the resulting chemiluminescence. cIEF electropherograms revealed both distinctive and overlapping peaks for Akt isoforms in all cell types (Figure 1A, Supplementary Figure S1A–J). In general, compared with unmodified Akt isoforms, Akt phosphoisoforms populate peaks at lower pI values [26]. Within each cell type, a single antibody directed against pan-Akt is sufficient to resolve all Akt isoforms [27]. However, the distribution and composition of Akt isoforms differed drastically across cell types (Supplementary Figure S1A–J). On the one hand, generation of individual cIEF electropherograms using antibodies directed against specific Akt isoforms was necessary to accurately distinguish associated peaks on the cIEF electropherograms generated using antibodies directed against pan-Akt. On the other hand, generation of cIEF electropherograms using antibodies directed against pan-Akt was necessary to evaluate the relative concentrations of Akt isoforms in each cell type. To ensure accurate and thorough analyses of Akt isoforms, all four antibodies (directed against pan-Akt, Akt1, Akt2, and Akt3) were used in all experiments described herein.

The cell types examined could be divided into two distinctive groups, namely, a group with Akt3 expression and a group without Akt3 expression (Table 1). Consistent with our previous observation, Akt3 was the dominant isoform in SH-SY5Y neuronal cells and accounted for approximately 50% of all Akt isoforms [19]. Akt1 and Akt2 accounted for approximately 40% and 10% of all Akt isoforms in SH-SY5Y neuronal cells, respectively (Figure 1A). In contrast, Akt3 was completely absent in HepG2 liver cells, whereas Akt1 was the dominant isoform, accounting for approximately 90% and Akt2 accounting for approximately 10% of all Akt isoforms (Figure 1B). Interestingly, differential expression of the Akt3 isoform was also observed within the evaluated immune cell populations. Akt3 accounted for 13% of all Akt isoforms in HMC3 microglial cells (Figure 1C), whereas Akt3 was completely absent in Jurkat T-lymphocytes (Figure 1D). The group of cells without Akt3 expression comprised of HepG2 liver cells, Jurkat T-lymphocytes, and Caco-2 intestinal cells. The group of cells with Akt3 expression comprised of SH-SY5Y neuronal cells (50%), HEK293 kidney cells (39%), subcutaneous pre-adipocytes (19%), MDA-MB-231 breast epithelial cells (18%), A549 lung epithelial cells (15%), HMC3 microglial cells (13%), and subcutaneous adipocytes (7%). Notably, the differentiation of subcutaneous pre-adipocytes into adipocytes was accompanied by a reduction in Akt3 expression of nearly three-fold.

### 2.2. Positive Regulation of the pI3K/Akt/mTOR Signaling Pathway in Akt3-Expressing Cells 

**Figure 2:**
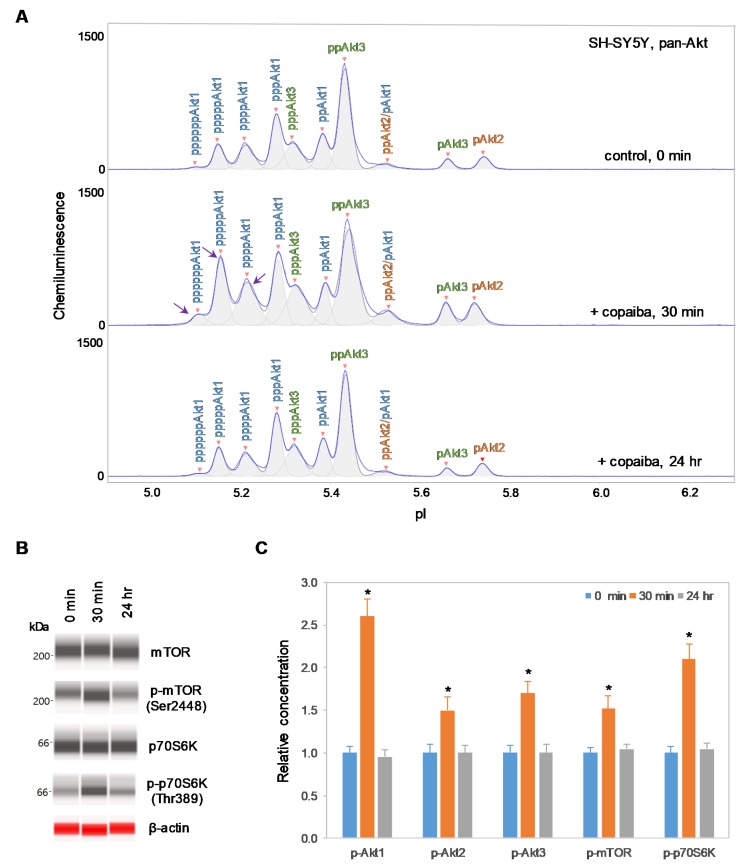
Positive regulation of the pI3K/Akt/mTOR signaling pathway in SH-SY5Y neuronal cells by copaiba essential oil. (**A**) cIEF electropherograms of pan-Akt in SH-SY5Y cells before (0 min) and after (30 min and 24 h) treatment with copaiba essential oil. Peaks associated with Akt isoforms are labeled and color-coded: Akt1 (blue), Akt2 (orange), and Akt3 (green). The purple arrows point to highly phosphorylated Akt1 peaks that transiently increased following treatment with copaiba essential oil. (**B**) Expression levels of the mTOR and p70S6K phosphoisoforms as a function of time after treatment with copaiba essential oil. β-Actin served as the loading control. (**C**) Relative concentrations of Akt1, Akt2, Akt3, mTOR, and p70S6K phosphoisoforms as a function of time after treatment with copaiba essential oil. The relative concentration describes the fold change in the expression level of a protein phosphoisoform after treatment compared to that under the control condition. The error bars indicate the standard deviations of six repeated measurements per experimental condition. The asterisks indicate a statistical significance of *p* ≤ 0.05 versus the control. SH-SY5Y cells were treated with 100 ng/mL copaiba essential oil.

**Figure 3:**
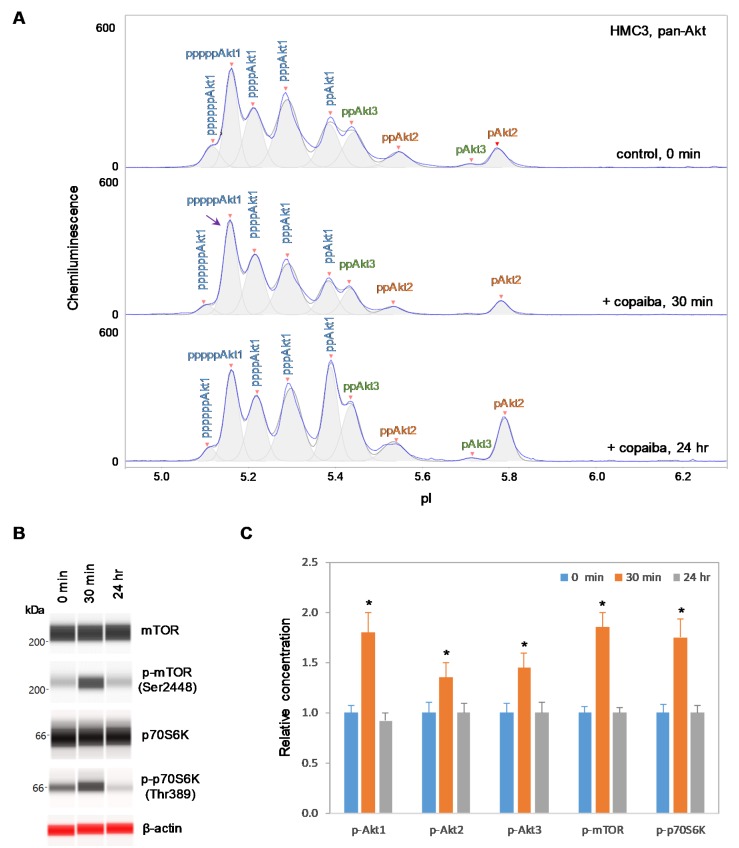
Positive regulation of the pI3K/Akt/mTOR signaling pathway in HMC3 microglial cells by copaiba essential oil. (**A**) cIEF electropherograms of pan-Akt in HMC3 cells before (0 min) and after (30 min and 24 h) treatment with copaiba essential oil. Peaks associated with Akt isoforms are labeled and color-coded: Akt1 (blue), Akt2 (orange), and Akt3 (green). The purple arrow points to the highly phosphorylated Akt1 peak that transiently increased following treatment with copaiba essential oil. Note the relative intensity of this peak compared to that of other peaks in the cIEF electropherogram. (**B**) Expression levels of the mTOR and p70S6K phosphoisoforms as a function of time after treatment with copaiba essential oil. β-Actin served as the loading control. (**C**) Relative concentrations of Akt1, Akt2, Akt3, mTOR, and p70S6K phosphoisoforms as a function of time after treatment with copaiba essential oil. The relative concentration describes the fold change in the expression level of a protein phosphoisoform after treatment compared to that under the control condition. The error bars indicate the standard deviations of six repeated measurements per experimental condition. The asterisks indicate a statistical significance of *p* ≤ 0.05 versus the control. HMC3 cells were treated with 100 ng/mL copaiba essential oil.

Interestingly, positive regulation of the pI3K/Akt/mTOR signaling pathway by copaiba essential oil was directly correlated with the expression of Akt3 isoforms. In SH-SY5Y neuronal cells and HMC3 microglial cells, which express Akt3, treatment with copaiba essential oil promoted transient phosphorylation of signaling proteins in the pI3K/Akt/mTOR signaling cascade (Figure 2 and Figure 3). Treatment with copaiba essential oil strongly increased the relative concentrations of highly phosphorylated Akt1 isoforms, which could be detected on the cIEF electropherograms using anti-pan-Akt primary antibodies (Figure 2A and Figure 3A). The positive regulatory effects of copaiba essential oil on the phosphorylation of Akt2 and Akt3 isoforms were much weaker, but were still detectable on cIEF electropherograms generated using anti-Akt2 or anti-Akt3 primary antibodies (Supplementary Figure S2). Furthermore, treatment with copaiba essential oil transiently increased the phosphorylation of mTOR and p70S6K proteins in SH-SY5Y neuronal cells (Figure 2B) and HMC3 microglial cells (Figure 3B). The expression levels of mTOR and p70S6K isoforms were measured via capillary western immunoassays in matrix-filled capillaries that separate proteins by size. In general, the positive regulatory effects of copaiba essential oil on the pI3K/Akt/mTOR signaling pathway were rapid and peaked at approximately 30 min post-treatment in cells that expressed Akt3 (Figure 2C and Figure 3C, Supplementary Figure S3).

### 2.3. Negative Regulation of the pI3K/Akt/mTOR Signaling Pathway in Cells Without Akt3 Expression

**Figure 4:**
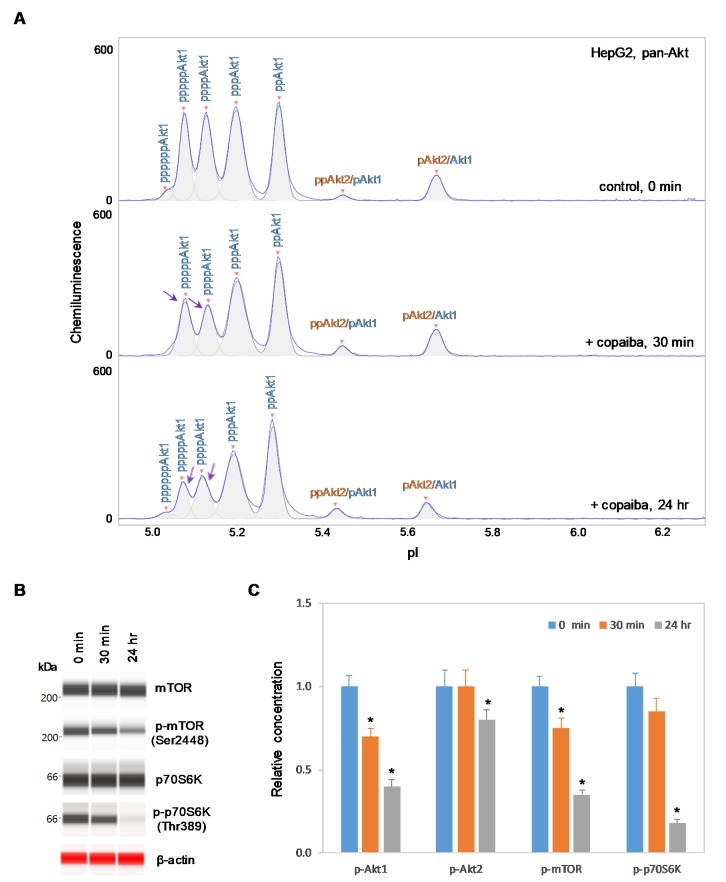
Negative regulation of the pI3K/Akt/mTOR signaling pathway in HepG2 liver cells by copaiba essential oil. (**A**) cIEF electropherograms of pan-Akt in HepG2 cells before (0 min) and after (30 min and 24 h) treatment with copaiba essential oil. Peaks associated with Akt isoforms are labeled and color-coded: Akt1 (blue) and Akt2 (orange). The purple arrows point to highly phosphorylated Akt1 peaks that exhibited a reduced amplitude following treatment with copaiba essential oil. (**B**) Expression levels of the mTOR and p70S6K phosphoisoforms as a function of time after treatment with copaiba essential oil. β-Actin serves as the loading control. (**C**) Relative concentrations of Akt1, Akt2, Akt3, mTOR, and p70S6K phosphoisoforms as a function of time after treatment with copaiba essential oil. The relative concentration describes the fold change in the expression level of a protein phosphoisoform after treatment compared to that under the control condition. The error bars indicate the standard deviations of six repeated measurements per experimental condition. The asterisks indicate a statistical significance of *p* ≤ 0.05 versus the control. HepG2 cells were treated with 100 ng/mL copaiba essential oil.

**Figure 5:**
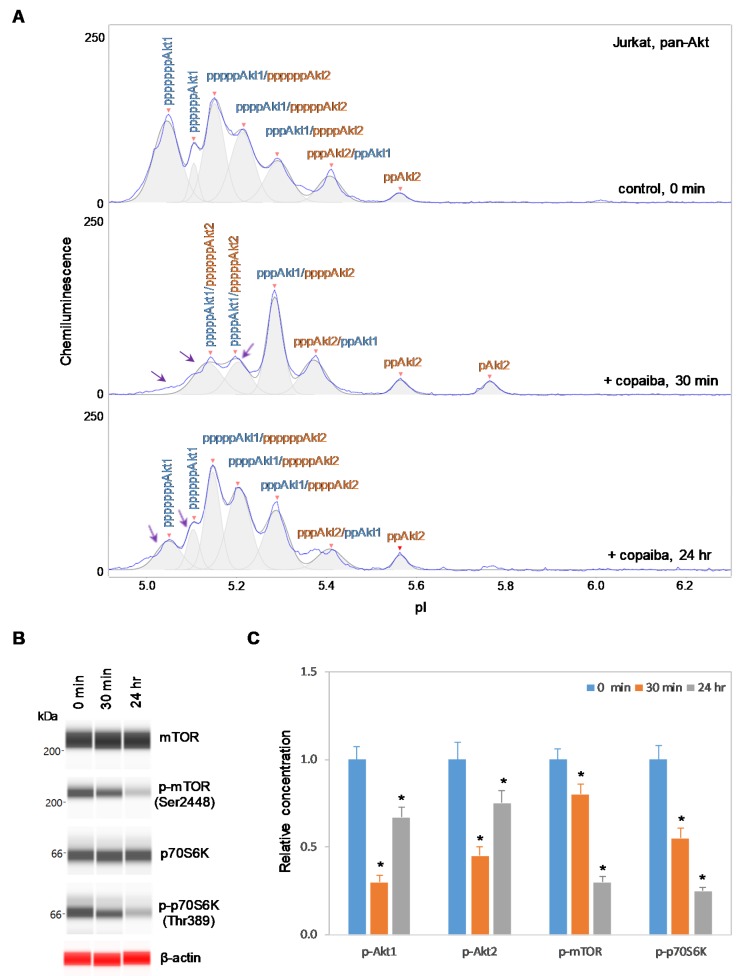
Negative regulation of the pI3K/Akt/mTOR signaling pathway in Jurkat T-cells by copaiba essential oil. (**A**) cIEF electropherograms of pan-Akt in Jurkat cells before (0 min) and after (30 min and 24 h) treatment with copaiba essential oil. Peaks associated with Akt isoforms are labeled and color-coded: Akt1 (blue) and Akt2 (orange). The purple arrows point to highly phosphorylated Akt1 peaks that exhibited a reduced amplitude following treatment with copaiba essential oil. (**B**) Expression levels of the mTOR and p70S6K phosphoisoforms as a function of time after treatment with copaiba essential oil. β-Actin serves as the loading control. (**C**) Relative concentrations of Akt1, Akt2, Akt3, mTOR, and p70S6K phosphoisoforms as a function of time after treatment with copaiba essential oil. The relative concentration describes the fold change in the expression level of a protein phosphoisoform after treatment compared to that under the control condition. The error bars indicate the standard deviations of six repeated measurements per experimental condition. The asterisks indicate a statistical significance of *p* ≤ 0.05 versus the control. Jurkat cells were treated with 100 ng/mL copaiba essential oil.

In contrast, negative regulation of the pI3K/Akt/mTOR signaling pathway by copaiba essential oil was observed in cells that lacked Akt3 expression. In HepG2 liver cells and Jurkat T-lymphocytes, which completely lacked Akt3 expression, treatment with copaiba essential oil progressively reduced the phosphorylation of signaling proteins in the pI3K/Akt/mTOR signaling cascade (Figure 4 and Figure 5). Treatment with copaiba essential oil progressively reduced the relative concentrations of highly phosphorylated Akt1 isoforms (Figure 4A and Figure 5A) as well as phosphorylated Akt2 isoforms (Supplementary Figure S4). Furthermore, treatment with copaiba essential oil progressively suppressed the phosphorylation of mTOR and p70S6K proteins in HepG2 liver cells (Figure 4B) and Jurkat T-lymphocytes (Figure 5B). In general, the negative regulatory effects of copaiba essential oil on the pI3K/Akt/mTOR signaling pathway were slow and remained noticeable at 24 h post-treatment in cells that lacked Akt3 expression (Figure 4C and Figure 5C, Supplementary Figure S3).

### 2.4. Expression of Akt3 Promotes Positive Regulation of the pI3K/Akt/mTOR Signaling Pathway

**Figure 6:**
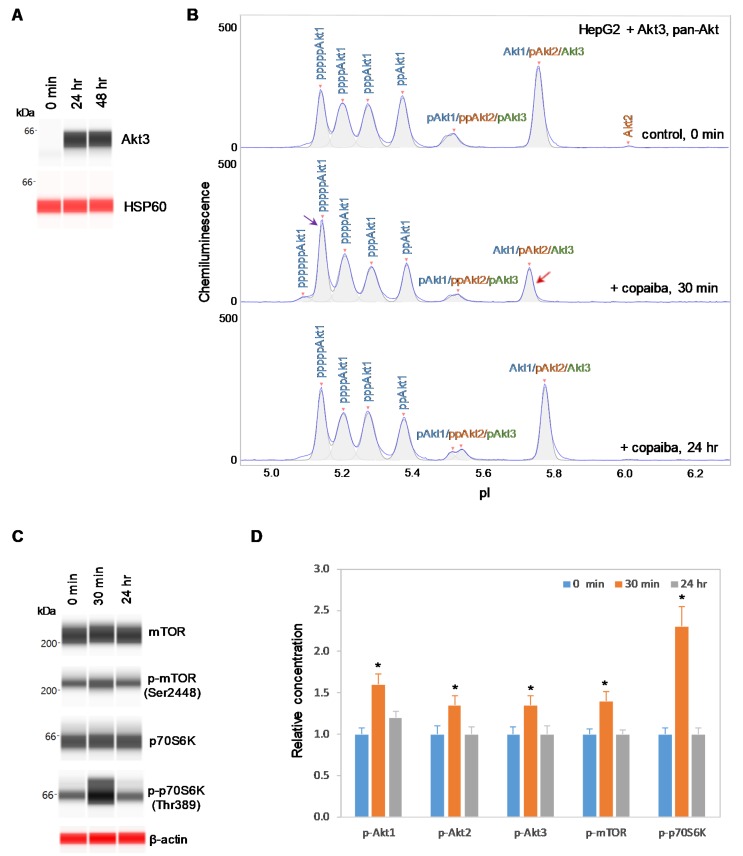
Expression of Akt3 in HepG2 cells promotes positive regulation of the pI3K/Akt/mTOR signaling pathway by copaiba essential oil. (**A**) Expression of Akt3 from plasmid DNA in HepG2 cells before (0 min) and after (24 h and 48 h) transfection with Akt3-encoding plasmid DNA. HSP60 served as the loading control. (**B**) cIEF electropherograms of pan-Akt in HepG2 cells transfected with Akt3-encoding plasmid DNA for 24 h as a function of time after treatment with copaiba essential oil. Peaks associated with Akt isoforms are labeled and color-coded: Akt1 (blue), Akt2 (orange), and Akt3 (green). The purple arrow points to the highly phosphorylated Akt1 peak that transiently increased following treatment with copaiba essential oil. Note the relative intensity of this peak compared to that of other peaks in the cIEF electropherogram. The red arrow points to the transient reduction in the relative intensity of a peak comprising the least-modified isoforms of Akt1, Akt2, and Akt3. (**C**) Expression levels of the mTOR and p70S6K phosphoisoforms as a function of time after treatment with copaiba essential oil. β-Actin served as the loading control. (**D**) Relative concentrations of Akt1, Akt2, Akt3, mTOR, and p70S6K phosphoisoforms as a function of time after treatment with copaiba essential oil. The relative concentration describes the fold change in the expression level of a protein phosphoisoform after treatment compared to that under the control condition. The error bars indicate the standard deviations of six repeated measurements per experimental condition. The asterisks indicate a statistical significance of *p* ≤ 0.05 versus the control. HepG2 cells were treated with 100 ng/mL copaiba essential oil.

To further examine the relationship between Akt3 and the pI3K/Akt/mTOR signaling pathway, HepG2 liver cells were transfected with an Akt3 expression plasmid. Akt3 expression, which was completely absent before transfection, was strongly detected at both 24 h and 48 h post-transfection in HepG2 cells, as measured with capillary western immunoassays (Figure 6A). Consistent with this result, the presence of Akt3 in HepG2 cells post-transfection was also detected with cIEF immunoassays (Supplementary Figure S5). Akt3 expressed from the plasmid DNA had higher pI values on the cIEF electropherograms than endogenous Akt3 due to the presence of a FLAG epitope. Most interestingly, treatment of transfected HepG2 cells with copaiba essential oil promoted transient phosphorylation of signaling proteins in the pI3K/Akt/mTOR signaling cascade (Figure 6B–D). Treatment with copaiba essential oil transiently increased the intensity of a peak associated with a highly phosphorylated Akt1 isoform at a low pI value, while concomitantly reducing the intensity of a peak associated with unmodified Akt1, unmodified Akt3, and monophosphorylated Akt2 at a high pI value (Figure 6B). The shifts of Akt isoforms toward lower pI values indicated an increase in phosphorylation activity. Furthermore, treatment with copaiba essential oil transiently increased the phosphorylation of mTOR and p70S6K proteins in transfected HepG2 cells. Clearly, the expression of Akt3 in HepG2 cells promoted positive regulation of the pI3K/Akt/mTOR signaling pathway in response to treatment with copaiba essential oil (Figure 6D). Notably, the positive regulatory effects of copaiba essential oil on the pI3K/Akt/mTOR signaling pathway were rapid and peaked at approximately 30 min post-treatment in HepG2 cells expressing Akt3.

### 2.5. Inhibition of Akt3 Expression Abrogates the Regulatory Effect on the pI3K/Akt/mTOR Signaling Pathway

**Figure 7:**
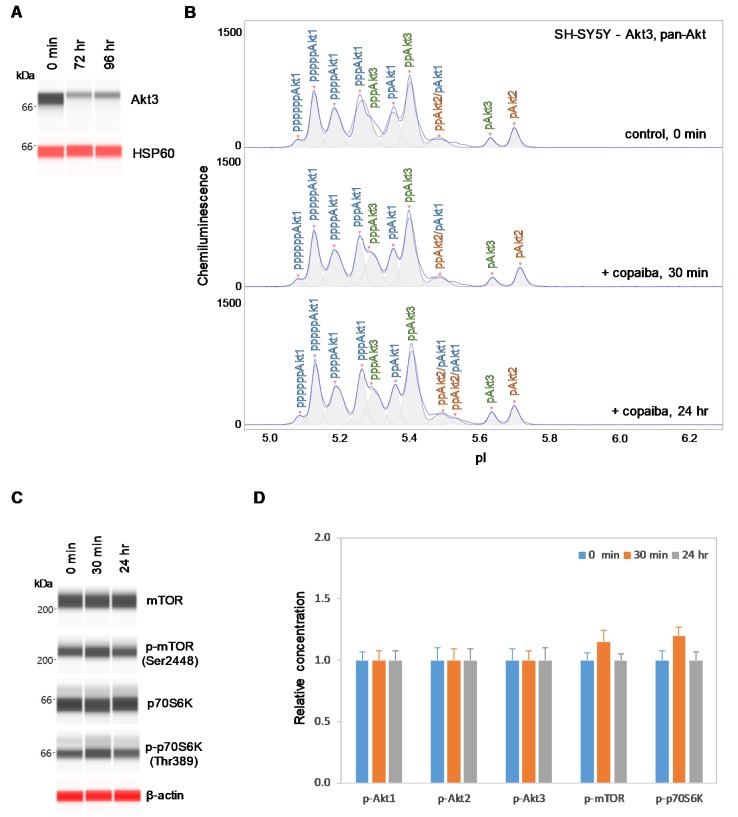
Suppression of Akt3 expression in SH-SY5Y cells reduces the positive regulation of the pI3K/Akt/mTOR signaling pathway by copaiba essential oil. (**A**) Expression levels of Akt3 in SH-SY5Y cells before (0 min) and after (24 h and 48 h) transfection with Akt3 siRNA. HSP60 served as the loading control. (**B**) cIEF electropherograms of pan-Akt in SH-SY5Y cells transfected with Akt3 siRNA for 24 h as a function of time after treatment with copaiba essential oil. Peaks associated with Akt isoforms are labeled and color-coded: Akt1 (blue), Akt2 (orange), and Akt3 (green). (**C**) Expression levels of the mTOR and p70S6K phosphoisoforms as a function of time after treatment with copaiba essential oil. β-Actin served as the loading control. (**D**) Relative concentrations of Akt1, Akt2, Akt3, mTOR, and p70S6K phosphoisoforms as a function of time after treatment with copaiba essential oil. The relative concentration describes the fold change in the expression level of a protein phosphoisoform after treatment compared to that under the control condition. The error bars indicate the standard deviations of six repeated measurements per experimental condition. SH-SY5Y cells were treated with 100 ng/mL copaiba essential oil.

Conversely, SH-SY5Y neuronal cells were transfected with small interfering RNA (siRNA) molecules targeting Akt3 transcripts. At 72 h and 96 h post-transfection, the Akt3 expression level was reduced by more than 80% compared to its pre-transfection level in SH-SY5Y cells, as measured with capillary western immunoassays (Figure 7A). Consistent with this finding, the relative intensity of peaks associated with Akt3 on the cIEF electropherograms was also substantially reduced compared to those associated with Akt1 and Akt2 (Figure 7B). Interestingly, treatment with copaiba essential oil had no detectable effect on signaling proteins in the pI3K/Akt/mTOR signaling pathway in SH-SY5Y cells with reduced Akt3 expression levels. Specifically, treatment with copaiba essential oil had no effect on the phosphorylation level of Akt1, Akt2, Akt3, mTOR, or p70S6K in SH-SY5Y cells transfected with siRNA molecules targeting Akt3 transcripts (Figure 7B–D). Thus, expression of Akt3 in SH-SY5Y cells was critical for the positive regulatory effects of copaiba essential oil on the pI3K/Akt/mTOR signaling pathway. It should be noted that approximately 20% of Akt3 was still detectable in SH-SY5Y cells treated with siRNA molecules targeting Akt3 transcripts. The difference in the regulatory effects of copaiba essential oil on the pI3K/Akt/mTOR signaling pathway between SH-SY5Y cells treated with siRNA molecules targeting Akt3 transcripts and HepG2 cells could be attributed to the reduced presence and complete absence of Akt3 in these cell lines, respectively.

### 2.6. Positive Regulation of the MAPK and JAK/STAT Signaling Pathways is Independent of Akt3 Expression

**Figure 8:**
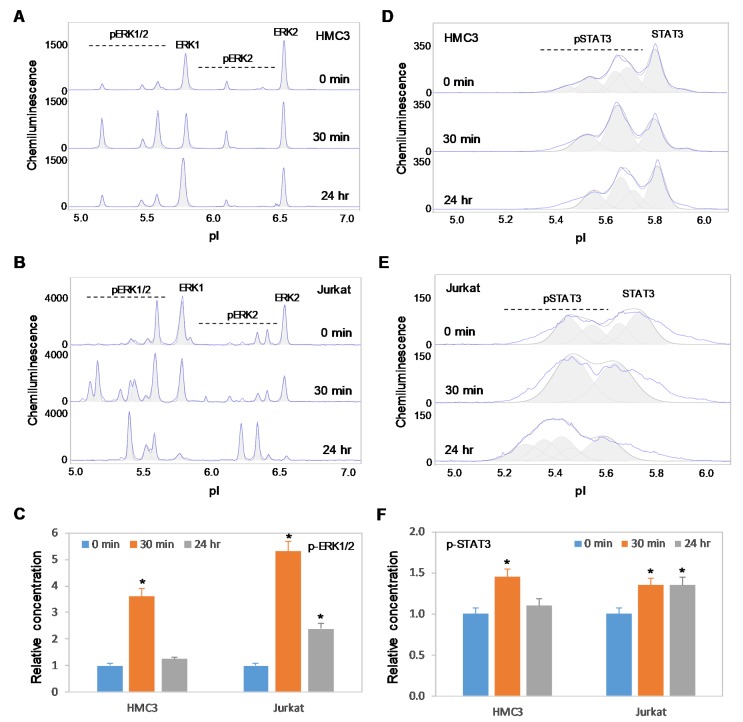
Positive regulation of the MAPK and JAK/STAT signaling pathways in immune cells by copaiba essential oils. (**A**,**B**) cIEF electropherograms of ERK1/2 in (A) HMC3 microglial cells and (B) Jurkat T-cells as a function of time after treatment with copaiba essential oil. (**C**) Relative concentrations of ERK1/2 phosphoisoforms as a function of time after treatment. (**D**,**E**) cIEF electropherograms of STAT in (D) HMC3 cells and (E) Jurkat cells as a function of time after treatment with copaiba essential oil. (**F**) Relative concentrations of STAT3 as a function of time after treatment. The relative concentration describes the fold change in the expression level of a protein phosphoisoform after treatment compared to that under the control condition. The error bars indicate the standard deviations of six repeated measurements per experimental condition. The asterisks indicate a statistical significance of *p* ≤ 0.05 versus the control. HMC3 and Jurkat cells were treated with 100 ng/mL copaiba essential oil.

**Figure 9:**
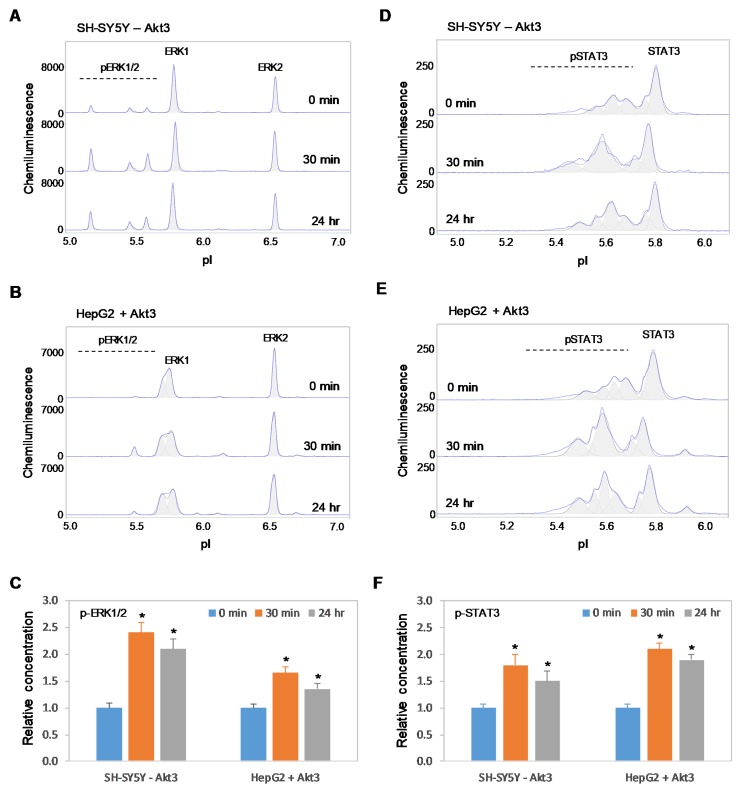
Positive regulation of the MAPK and JAK/STAT signaling pathways by copaiba essential oil is independent of Akt3 expression. (**A**,**B**) cIEF electropherograms of ERK1/2 in (A) SH-SY5Y cells transfected with siRNA molecules that target Akt3 transcripts and (B) HepG2 cells that express Akt3 from plasmid DNA as a function of time after treatment with copaiba essential oil. (**C**) Relative concentrations of ERK1/2 phosphoisoforms as a function of time after treatment. (**D**,**E**) cIEF electropherograms of STAT in (D) SH-SY5Y cells transfected with Akt3 siRNA and (E) HepG2 cells transfected with Akt3-encoding plasmid DNA as a function of time after treatment with copaiba essential oil. (**F**) Relative concentrations of STAT3 as a function of time after treatment. The relative concentration describes the fold change in the expression level of a protein phosphoisoform after treatment compared to that under the control condition. The error bars indicate the standard deviations of six repeated measurements per experimental condition. The asterisks indicate a statistical significance of *p* ≤ 0.05 versus the control. SH-SY5Y and HepG2 cells were treated with 100 ng/mL copaiba essential oil.

Previously, we reported the positive regulatory effects on the MAPK and JAK/STAT signaling pathways by copaiba essential oil in both SH-SY5Y neuronal cells and HepG2 liver cells [18]. In this study, we found that copaiba essential oil positively regulated the MAPK and JAK/STAT signaling pathways in both HMC3 microglial cells and Jurkat T-lymphocytes (Figure 8A–F). In addition, copaiba essential oil positively regulated the MAPK and JAK/STAT signaling pathways in SH-SY5Y cells transfected with siRNA molecules targeting Akt3 transcripts, as well as in HepG2 cells transfected with plasmid DNA encoding Akt3 (Figure 9A–F). Independent of the cell type or Akt3 expression level, treatment with copaiba essential oil consistently increased the phosphorylation of ERK1/2 and STAT3, which are biomarkers for the MAPK and JAK/STAT signaling pathways, respectively. Collectively, these data revealed that the positive regulatory effects of copaiba essential oil on the MAPK and JAK/STAT signaling pathways were independent of Akt3 expression.

## 3. Discussion

In this study, we reported the tissue-specific expression of Akt3 in cultured cells of various tissue origins. Our observation is consistent with the existing literature [23]. The expression of Akt3 was highest in neuronal and kidney cells and undetectable in liver and intestinal cells. Even within immune cell populations, microglial cells express Akt3, while T lymphocytes lack Akt3. Interestingly, the presence of Akt3 in neuronal and microglial cells was associated with positive regulatory effects of copaiba essential oil on the pI3K/Akt/mTOR signaling pathway. Conversely, the absence of Akt3 in liver cells and T lymphocytes was associated with negative regulatory effects of copaiba essential oil on the pI3K/Akt/mTOR signaling pathway. The expression of Akt3 via plasmid DNA in liver cells resulted in positive regulatory effects of copaiba essential oil on the pI3K/Akt/mTOR signaling pathway. In contrast, inhibition of Akt3 expression via siRNA in neuronal cells abrogated the regulatory effects of copaiba essential oil on the pI3K/Akt/mTOR signaling pathway. The Akt3 expression level had no impact on the positive regulatory effects of copaiba essential oil on the MAPK and JAK/STAT signaling pathways. Collectively, these data indicated that Akt3 was responsible for the positive regulatory effects of copaiba essential oil on the pI3K/Akt/mTOR signaling pathway.

The tissue-specific expression of Akt3 and the differential regulation of the pI3K/Akt/mTOR signaling pathway in response to copaiba essential oil suggest that the route of administration matters. For example, topical application of copaiba essential oil likely affects subcutaneous tissues, with positive regulation of the pI3K/Akt/mTOR signaling pathway in pre-adipocytes and adipocytes. Aromatherapy inhalation of copaiba essential oil likely affects lung and neuronal tissues, with positive regulation of the pI3K/Akt/mTOR signaling pathway. In contrast, ingestion of copaiba essential oil encapsulated in soft gels likely affects intestinal and, subsequently, liver cells, with negative regulation of the pI3K/Akt/mTOR signaling pathway. Currently, copaiba essential oil is used primarily for its purported pain relief and anti-inflammatory benefits. However, regulation of the JAK/STAT signaling pathway is independent of Akt3 expression. In fact, copaiba essential oil consistently upregulated the JAK/STAT signaling pathway in all evaluated cell types. Thus, tissue-specific expression of Akt3 may have no impact on the regulatory effects of copaiba essential oil on cell immunity. However, regulation of immunity and metabolic responses in mammals is highly coordinated and integrated [28,29]. Disruption of the metabolism-immunity interface underlies many chronic metabolic diseases, such as type 2 diabetes, atherosclerosis, and neurodegenerative disorders [30,31]. The regulatory effects of copaiba essential oil on the immune response may also be dependent on the route of administration, although further investigation is warranted.

The Akt3-mediated regulation of the tissue-specific response further links copaiba essential oil to neuronal signaling activities associated with inflammation and neuropathic pain. Akt3 is the dominant isoform in brain tissues, as described in previously published literature [32], and in neuronal cells, as described herein. The expression of Akt3 is critical for normal brain size and development [32,33]. The loss of Akt3 function induces depressive and anxiety-like behaviors [34] as well as learning and memory deficits [35]. In addition, Akt3 inhibits adipogenesis and protects against diet-induced obesity [36]. Consistently, we observed an approximately three-fold downregulation of Akt3 expression in subcutaneous adipocytes compared to pre-adipocytes (Table 1). Akt3 directly phosphorylates lysine-deficient protein kinase-1 (WNK1) and promotes its degradation in adipocytes [36]. Interestingly, WNK1 signaling regulates renal epithelial transport, ion and cell volume homeostasis, and γ-aminobutyric acid (GABA) signaling—processes that are relevant for various pathologic conditions such as hypertension, cerebral edema, and neuropathic pain [37]. On the one hand, Akt3 regulates the effects of copaiba essential oil on the pI3K/Akt/mTOR signaling pathway, which is critical for neuronal physiology, plasticity, metabolism, and homeostasis [38]. On the other hand, copaiba essential oil directly interacts with CB2 [3], which is a therapeutic target for inflammatory and neuropathic pain [39]. Future studies that examine the intricate relationships between copaiba essential oil, the metabolism–immunity interface, CB2-mediated signaling pathways, and the Akt3/WNK signaling pathway should further illuminate the mechanisms underlying the therapeutic potential of copaiba essential oil for the prevention of pain and inflammation. 

## 4. Materials and Methods

### 4.1. Cell Lines and Culture Conditions

Cell lines were purchased from the American Type Culture Collection (ATCC, Manassas, VA, USA) and cultured using ATCC-recommended protocols and culture media. Subcutaneous pre-adipocytes and adipocytes were purchased from Zen-Bio (Research Triangle Park, NC, USA) and maintained according to the manufacturer’s protocols. Subcutaneous pre-adipocytes were collected from a single donor who was undergoing elective surgery. Subcutaneous adipocytes were differentiated in vitro from pre-adipocytes for two weeks. Cell lines and culture media are listed in Supplementary Table S1.

### 4.2. Cell Treatment Conditions

Cell lines were grown in culture media to a confluence of approximately 70%. Culture media were replaced with culture media premixed with 100 ng/mL copaiba essential oil. This concentration was chosen to be close to the half-maximal effective concentration (EC_50_) of copaiba essential oil, which was previously determined to be approximately 80 ng/mL in SH-SY5Y cells [20]. The incubation time in the new culture media was considered as the time after treatment. 

### 4.3. Copaiba Essential Oil 

Copaiba essential oil (batch no. 180437) was obtained from dōTERRA International (Pleasant Grove, UT, USA). Chemical composition analysis of this specific copaiba essential oil using gas chromatography coupled to mass spectrometry has been previously described [20]. Eleven major terpenes and their percentages are as follows: β-caryophyllene (45.24%), α-copaene (7.19%), α-bergamotene (7.19%), α-humulene (7.18%), germacrene D (5.16%), β-bisabolene (3.47%), Δ-cadinene (2.78%), γ-elemene (2.26%), trans-cadina-1(6),4-diene (2.03%), β-elemene (1.67%), and α-cubebene (1.51%). 

### 4.4. Akt3 Expression via Plasmid DNA in HepG2 Cells

Approximately 4 µg of Akt3 expression plasmid (cat. no. RC224750, Origene, Rockville, MD, USA) in 500 µL of reduced serum medium (cat. no. 31985062, ThermoFisher Scientific, Waltham, MA, USA) was added to 12 µL of DNA transfection reagent (cat. no. 06366236001, Roche, Basel, Switzerland) and incubated for 30 min at room temperature. The transfection reagent–plasmid DNA complex was added in a dropwise manner to approximately 1 million HepG2 cells in a 6-cm culture dish with 4 mL of growth medium and incubated for 24 h. Transfected HepG2 cells were either collected and assayed for the Akt3 expression level or used for treatment with copaiba essential oil.

### 4.5. Inhibition of Akt3 Expression via siRNA in SH-SY5Y Cells

Akt3 siRNA (approximately 40 picomolar; cat. no. 110901, ThermoFisher Scientific) in 300 µL of reduced serum medium was mixed together with 18 µL of RNAi transfection reagent (cat. no. 13778075, Thermo Fisher Scientific) in 300 µL of reduced-serum medium and incubated for 5 min at room temperature. The transfection reagent–siRNA complex was added in a dropwise manner to approximately 2 million SH-SY5Y cells in a 6-cm culture dish with 4 mL of growth medium and incubated for 72 h. Transfected SH-SY5Y cells were either collected and assayed for the Akt3 expression level or used for treatment with copaiba essential oil.

### 4.6. Preparation of Cell Lysates

Approximately one million cells were incubated on ice for 10 min with 60 µL of lysis buffer (cat. no. 040-764, ProteinSimple, Santa Clara, CA, USA), sonicated 4 times for 5 s each, mixed by rotation for 2 h at 4 °C, and centrifuged at 12,000 rpm in an Eppendorf 5430R microfuge for 20 min at 4 °C. The supernatant was collected as the cell lysate. The total protein concentration in the cell lysate was determined with a Bradford protein assay and adjusted to a final concentration of 0.3 µg/µL with separation gradients (cat. no. Premix G2, pH 5–8, ProteinSimple) for charge-based cIEF immunoassays or 0.4 µg/µL with denaturing buffers (cat. no. PS-ST01EZ or PS-ST03EZ, ProteinSimple) for size-based western immunoassays.

### 4.7. cIEF Immunoassays

Cell lysates in separation gradients were loaded into 384-well assay plates (cat. no. 040-663, ProteinSimple) preloaded with primary and secondary antibodies and chemiluminescent substrates. Charge-based protein separation and detection in individual capillaries were performed using the default protocols of the NanoPro 1000 system (ProteinSimple). Hsp70 was used as the loading control. All cIEF immunoassays were performed in triplicate for each protein, and duplicate experiments were performed for each treatment condition, producing six repeated measurements per protein.

### 4.8. Capillary Western Immunoassays

Cell lysates in denaturing buffers were denatured at 95 °C for 5 min, and then transferred to assay plates (cat. no. SM-W004 or SM-W008, ProteinSimple) preloaded with blocking reagents, wash buffer, primary and secondary antibodies, and chemiluminescent substrates. Sized-based protein separation and detection in capillaries were performed using the default protocols of the Jess system (ProteinSimple). β-Actin and HSP60 were used as loading controls. All capillary western immunoassays were performed in triplicate for each protein, and duplicate experiments were performed for each treatment condition, producing six repeated measurements per protein.

### 4.9. Antibodies and Biomarker Proteins

The primary and secondary antibodies used are listed in Supplementary Table S2. The functions of biomarker proteins are listed in Supplementary Table S3.

### 4.10. Data Analysis

The assignment of pI values to protein phosphoisoforms was based on previously published literature [26,27,40,41,42,43,44,45], our own published data [18,19,20,46,47,48,49,50], and data provided herein. Protein expression levels were quantified using the Compass software from ProteinSimple. The expression levels of protein phosphoisoforms were normalized with respect to those of the loading controls for both charge-based and size-based immunoassays and further normalized to the total protein expression levels for size-based immunoassays.

### 4.11. Statistical Analysis

The data are presented as the mean values ± standard deviations across six repeated measurements. Statistical significance was calculated with Student’s t-test and thresholded at p ≤ 0.05 versus the control.

## Figures and Tables

**Table 1:** Composition of Akt isoforms in cells of various origins.

Cell Type	Origin	Disease	Cat. No.	Akt1 (%)	Akt2 (%)	Akt3 (%)
SH-SY5Y	Bone marrow	Neuroblastoma	CRL-2266, ATCC	40 ± 3	10 ± 1	50 ± 5
HepG2	Liver	Hepatocellular carcinoma	HB-8065, ATCC	86 ± 4	14 ± 5	0 ± 0
HMC3	Brain, microglia	Neuroinflammation	CRL-3304, ATCC	75 ± 7	12 ± 1	13 ± 1
Jurkat	Peripheral blood	Acute T-cell leukemia	TIB-152, ATCC	55 ± 6	45 ± 2	0 ± 0
Caco-2	Colon	Colorectal adenocarcinoma	HTB-37, ATCC	46 ± 4	54 ± 5	0 ± 0
A549	Lung	Lung carcinoma	CCL-185, ATCC	28 ± 2	57 ± 5	15 ± 1
HEK293	Kidney	Normal embryonic kidney	CRL-1573, ATCC	37 ± 3	24 ± 2	39 ± 3
MDA-MB-231	Breast	Mammary adenocarcinoma	CRM-HTB-26, ATCC	71 ± 6	11 ± 1	18 ± 2
Pre-adipocytes	Subcutaneous adipose tissue	Obesity	SP-2006-3, Zen-Bio	57 ± 5	23 ± 2	19 ± 2
Adipocytes	Subcutaneous adipose tissue	Obesity	SA-1006-3, Zen-Bio	58 ± 5	35 ± 3	7 ± 1

## Supplementary Materials

The following materials are available online at https://www.mdpi.com/1422-0067/21/8/2851/s1, Supplementary Figure S1, Akt isoform identification using capillary isoelectric focusing immunoassays; Supplementary Figure S2, Copaiba essential oil treatment transiently increases the phosphorylation of Akt1, Akt2, and Akt3 in HMC3 microglial cells; Supplementary Figure S3, Time-dependent regulation of Akt1 phosphorylation by copaiba essential oil in SH-SY5Y versus HepG2 cells; Supplementary Figure S4, Copaiba essential oil treatment reduces the phosphorylation of Akt1 and Akt2 in Jurkat T-lymphocytes; Supplementary Figure S5, Akt isoform identification in HepG2 cells transfected with Akt3-encoding plasmid DNA; Supplementary Table S1, List of culture media; Supplementary Table S2, List of primary and secondary antibodies; Supplementary Table S3, List of biomarker proteins and their functions.
