# Supplementary material for: Akt3 Regulates the Tissue-Specific Response to Copaiba Essential Oil
Source: Int J Mol Sci. 2020 Apr 19;21(8):2851. doi: 10.3390/ijms21082851 (PMC7216139; doi:10.3390/ijms21082851)
Supplement: Supplementary file 1 [file ijms-21-02851-s001.pdf]

Supplementary Materials for

Akt3 Regulates the Tissue-Specific Response to Copaiba Essential Oil

Yasuyo Urasaki[1], Cody Beaumont[2], Jeffery N. Talbot[1], David K. Hill[2], Thuc T. Le[1,*]

[1]College of Pharmacy, Roseman University of Health Sciences, 10530 Discovery Drive, Las Vegas, NV 89135, USA.

[2]dōTERRA International, LLC, 389 South 1300 West, Pleasant Grove, Utah 84062, USA.

*To whom correspondence should be addressed: Email: tle5@roseman.edu; Tel: 1-702-802-2820

A

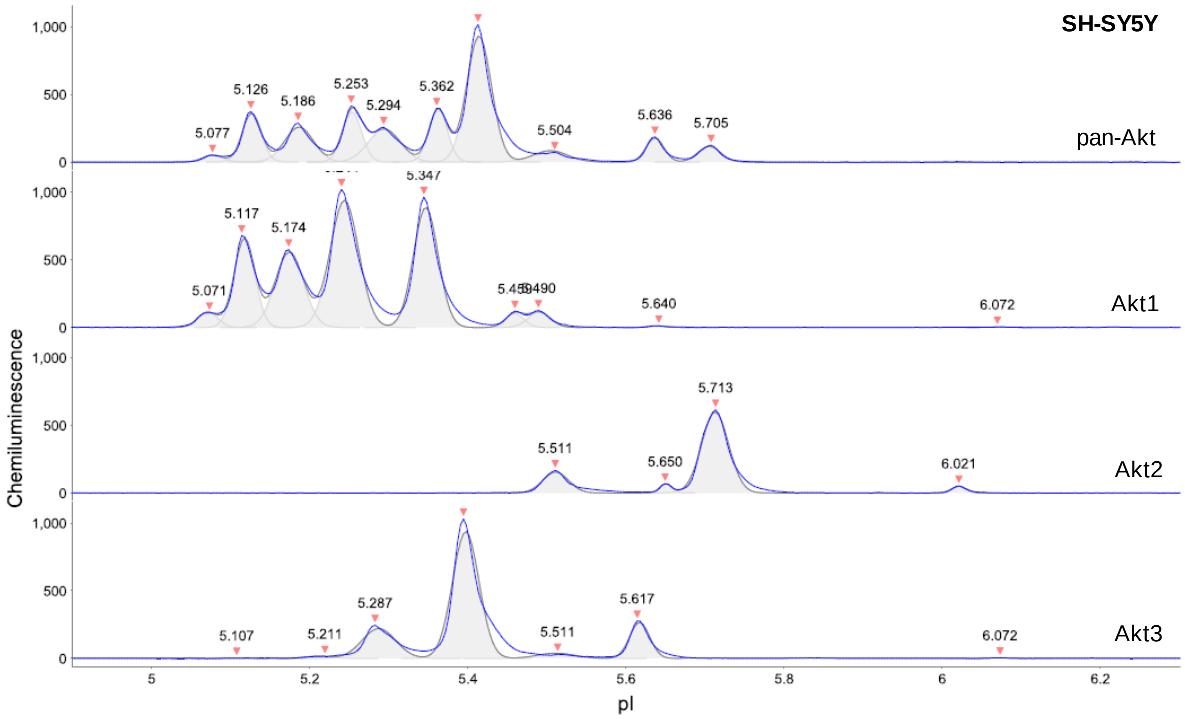
SH-SY5Y
pan-Akt
Akt1
Akt2
Akt3
Chemiluminescence
pI
1,000
500
0
5
5.2
5.4
5.6
5.8
6
6.2
5.077
5.126
5.186
5.253
5.294
5.362
5.504
5.636
5.705
5.071
5.117
5.174
5.347
5.640
6.072
5.511
5.650
5.713
6.021
5.107
5.211
5.287
5.511
5.617
6.072

B

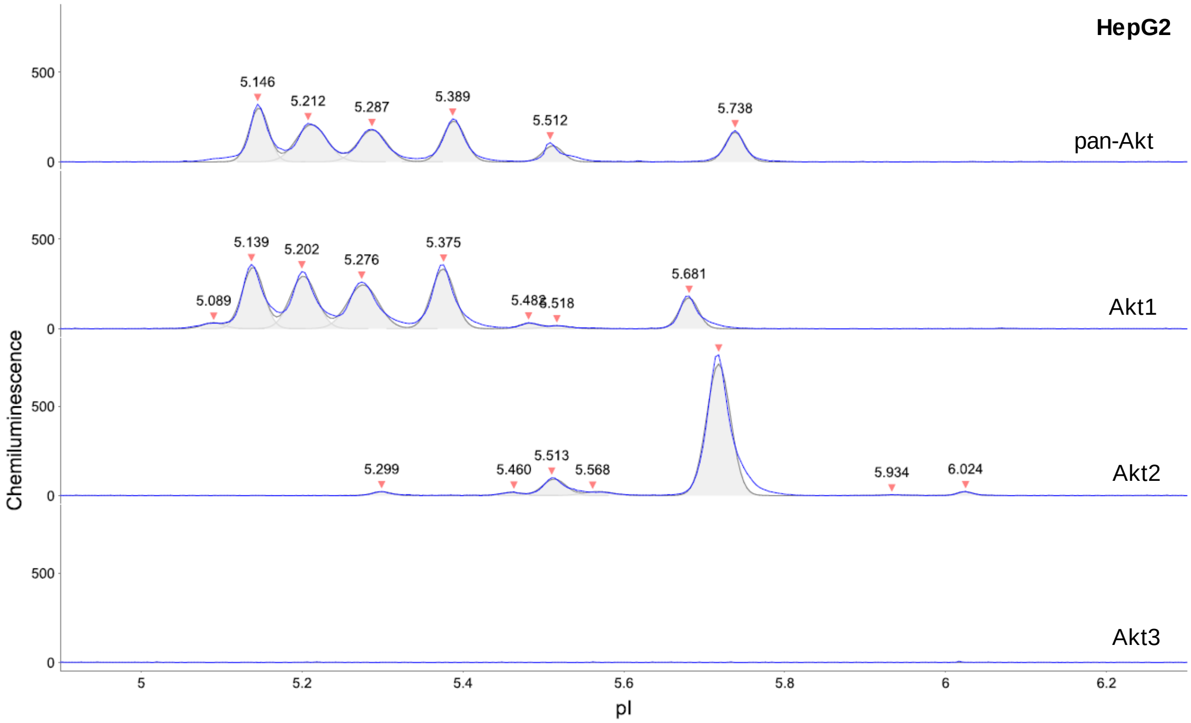
HepG2
pan-Akt
Akt1
Akt2
Akt3
Chemiluminescence
pI
500
0
5
5.2
5.4
5.6
5.8
6
6.2
5.146
5.212
5.287
5.389
5.512
5.738
5.089
5.139
5.202
5.276
5.375
5.518
5.681
5.299
5.460
5.513
5.568
5.934
6.024

C

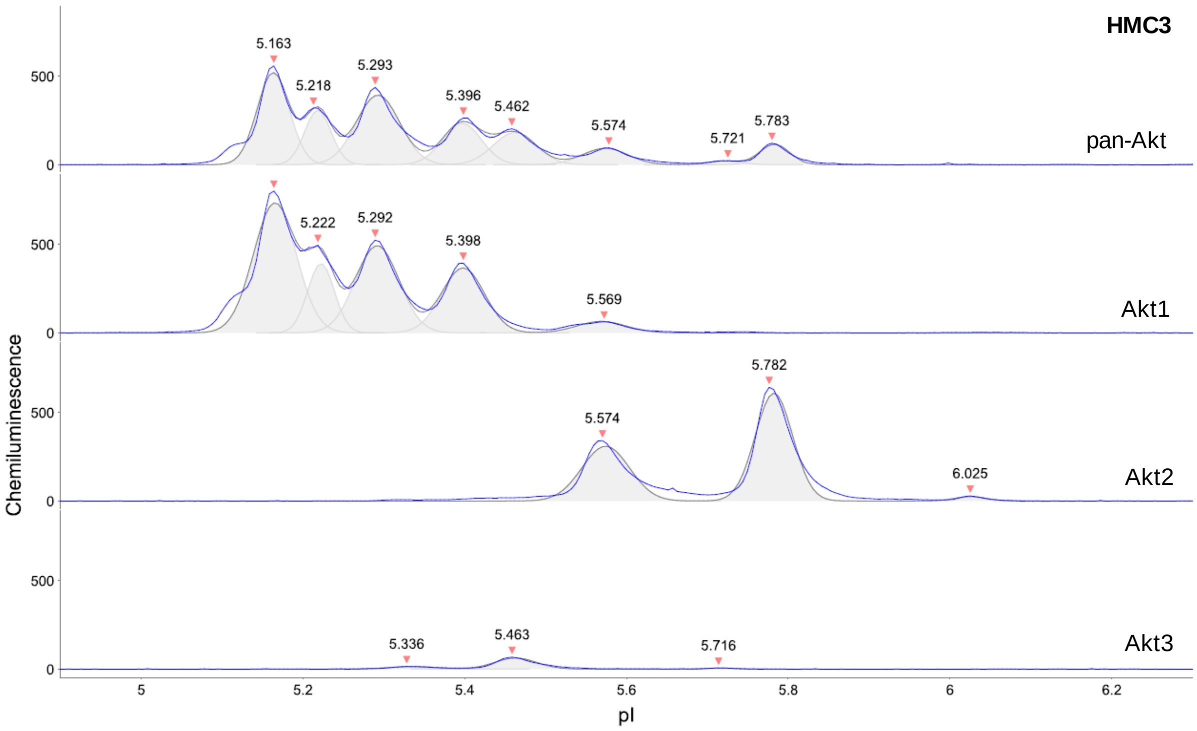
HMC3
pan-Akt
5.163
5.218
5.293
5.396
5.462
5.574
5.721
5.783
Akt1
5.222
5.292
5.398
5.569
Akt2
5.574
5.782
6.025
Akt3
5.336
5.463
5.716
Chemiluminescence
500
0
5
5.2
5.4
5.6
5.8
6
6.2
pI

D

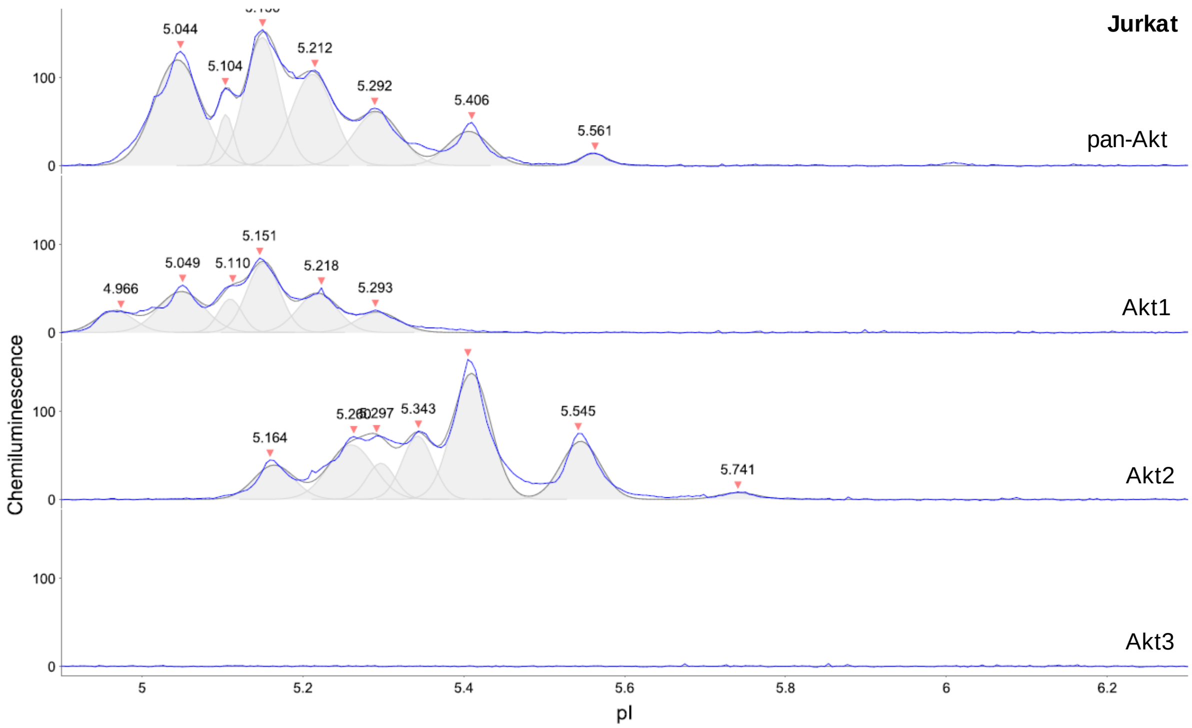
Jurkat
pan-Akt
5.044
5.104
5.212
5.292
5.406
5.561
Akt1
4.966
5.049
5.110
5.151
5.218
5.293
Akt2
5.164
5.297
5.343
5.545
5.741
Akt3
Chemiluminescence
100
0
5
5.2
5.4
5.6
5.8
6
6.2
pI

E

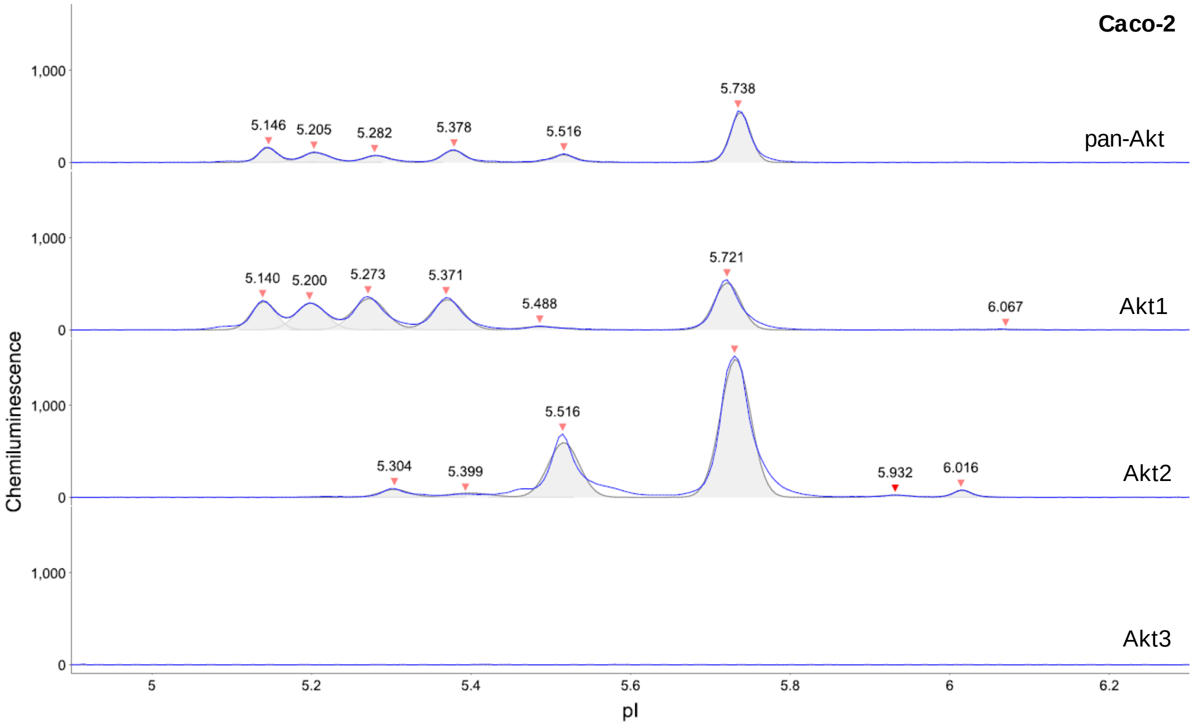

Caco-2
pan-Akt
Akt1
Akt2
Akt3
Chemiluminescence
1,000
0
5.146
5.205
5.282
5.378
5.516
5.738
5.140
5.200
5.273
5.371
5.488
5.721
6.067
5.304
5.399
5.516
5.932
6.016
5
5.2
5.4
5.6
5.8
6
6.2
pI

F

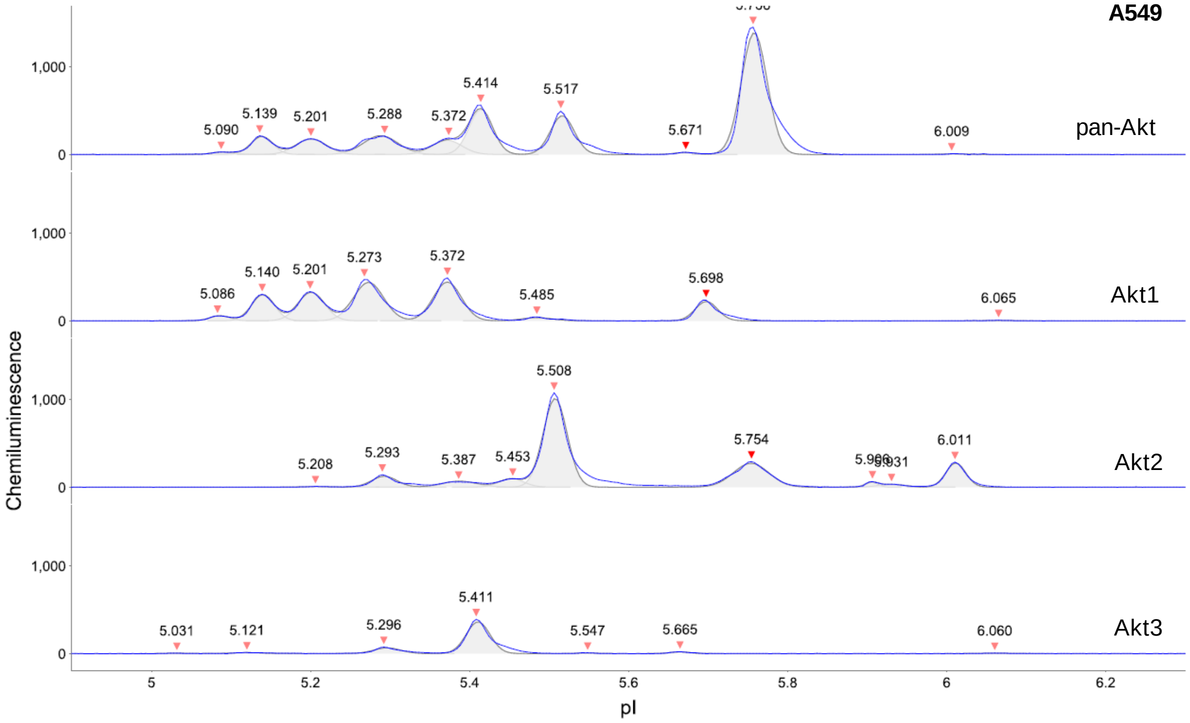

A549
pan-Akt
Akt1
Akt2
Akt3
Chemiluminescence
1,000
0
5.090
5.139
5.201
5.288
5.372
5.414
5.517
5.671
6.009
5.086
5.140
5.201
5.273
5.372
5.485
5.698
6.065
5.208
5.293
5.387
5.453
5.508
5.754
6.011
5.031
5.121
5.296
5.411
5.547
5.665
6.060
5
5.2
5.4
5.6
5.8
6
6.2
pI

G

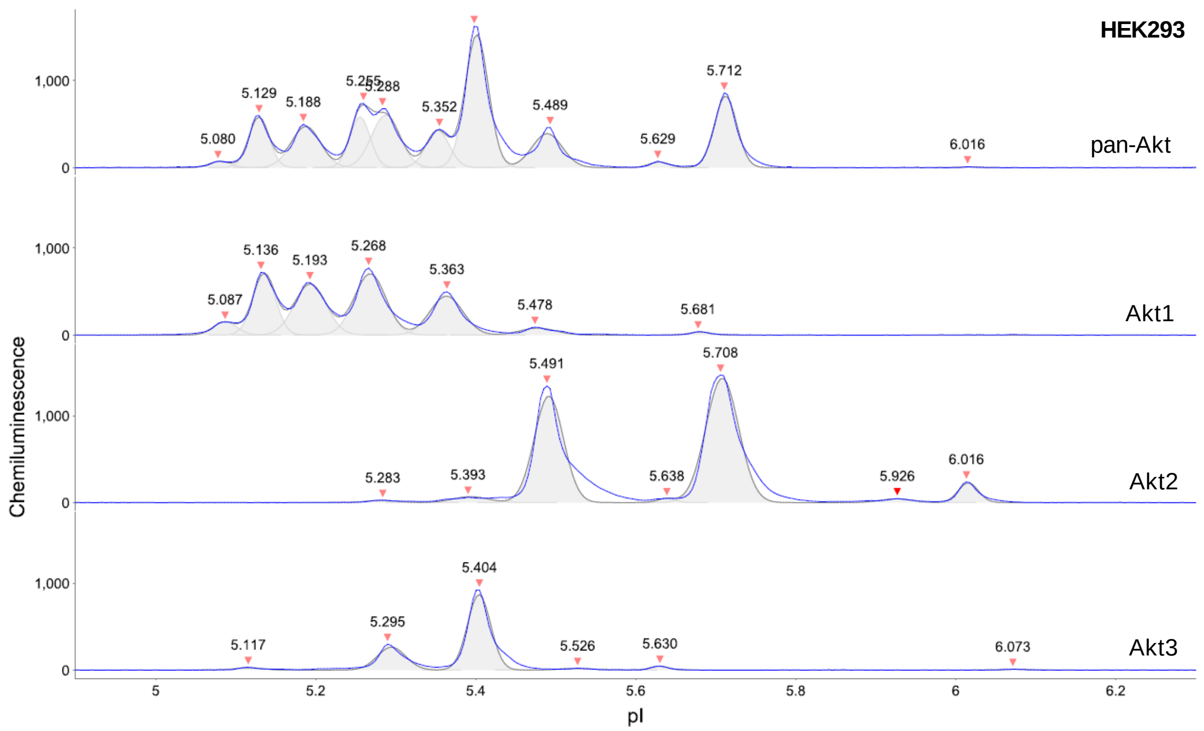
HEK293
pan-Akt
Akt1
Akt2
Akt3
Chemiluminescence
pI
5.080
5.129
5.188
5.255
5.288
5.352
5.489
5.629
5.712
6.016
5.087
5.136
5.193
5.268
5.363
5.478
5.681
5.283
5.393
5.491
5.638
5.708
5.926
6.016
5.117
5.295
5.404
5.526
5.630
6.073

H

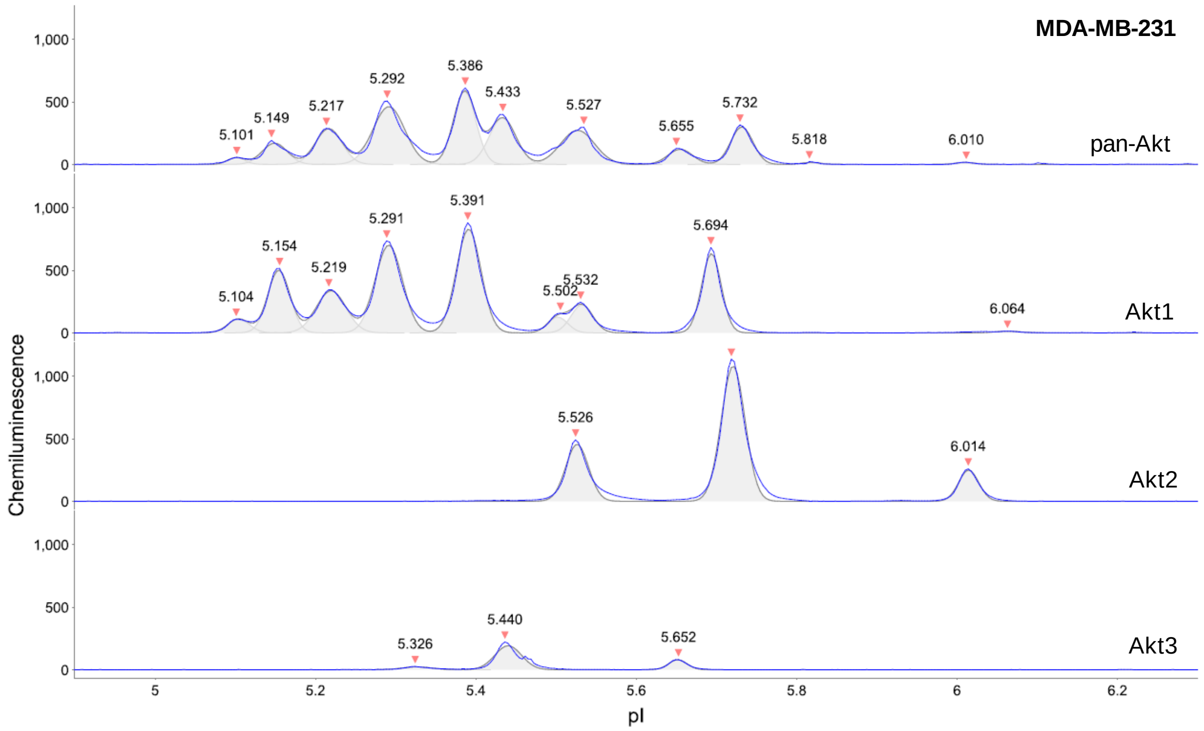
MDA-MB-231
pan-Akt
Akt1
Akt2
Akt3
Chemiluminescence
pI
5.101
5.149
5.217
5.292
5.386
5.433
5.527
5.655
5.732
5.818
6.010
5.104
5.154
5.219
5.291
5.391
5.502
5.532
5.694
6.064
5.526
6.014
5.326
5.440
5.652

I

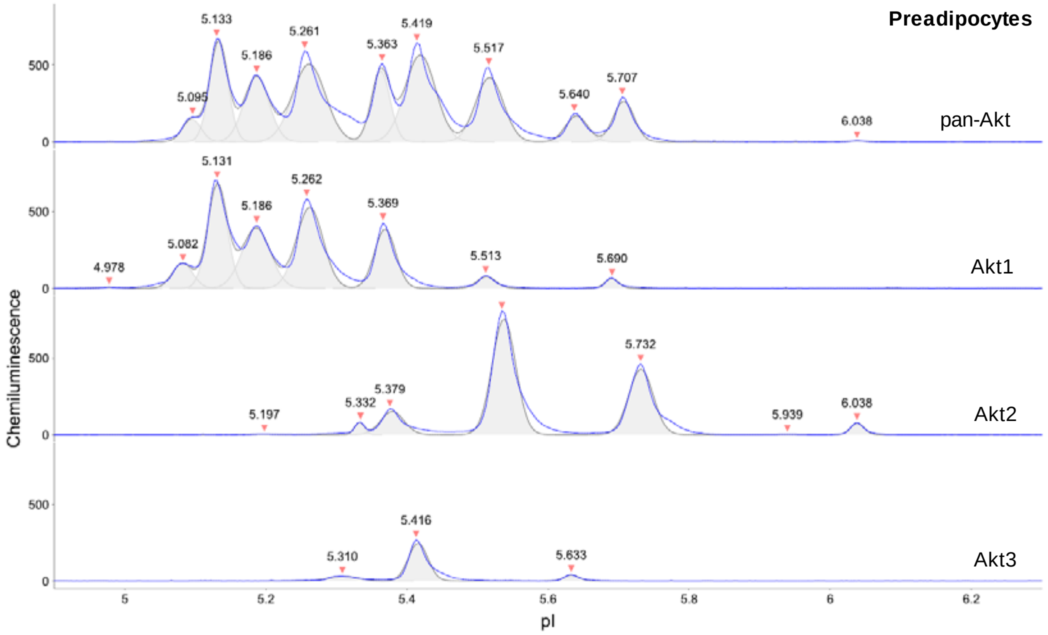

J

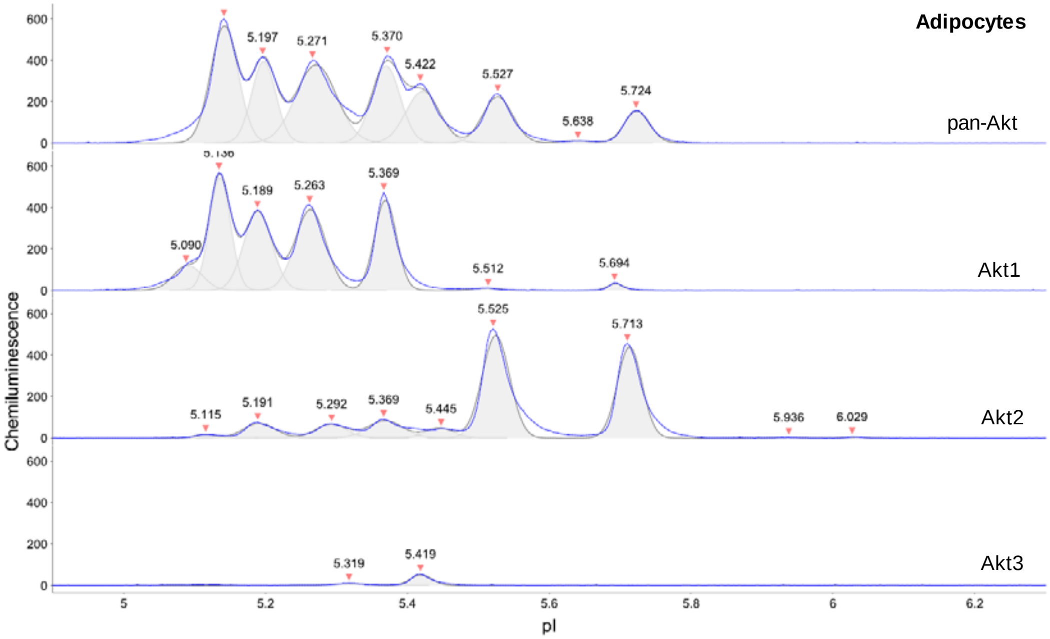

Supplementary Figure S1. Akt isoform identification using capillary isoelectric focusing (cIEF) immunoassays. Primary antibodies against pan-Akt (top panel), Akt1 (second panel), Akt2 (third panel), and Akt3 (fourth panel) were used. cIEF electropherograms of pan-Akt, Akt1, Akt2, and Akt3 in (A) SH-SY5Y neuronal cells, (B) HepG2 liver cells, (C) HMC3 microglial cells, (D) Jurkat T cells, (E) Caco-2 intestinal cells, (F) A549 lung cells, (G) HEK293 kidney cells, (H) MDA-MB-231 breast epithelial cells, (I) subcutaneous preadipocytes, and (J) subcutaneous adipocytes.

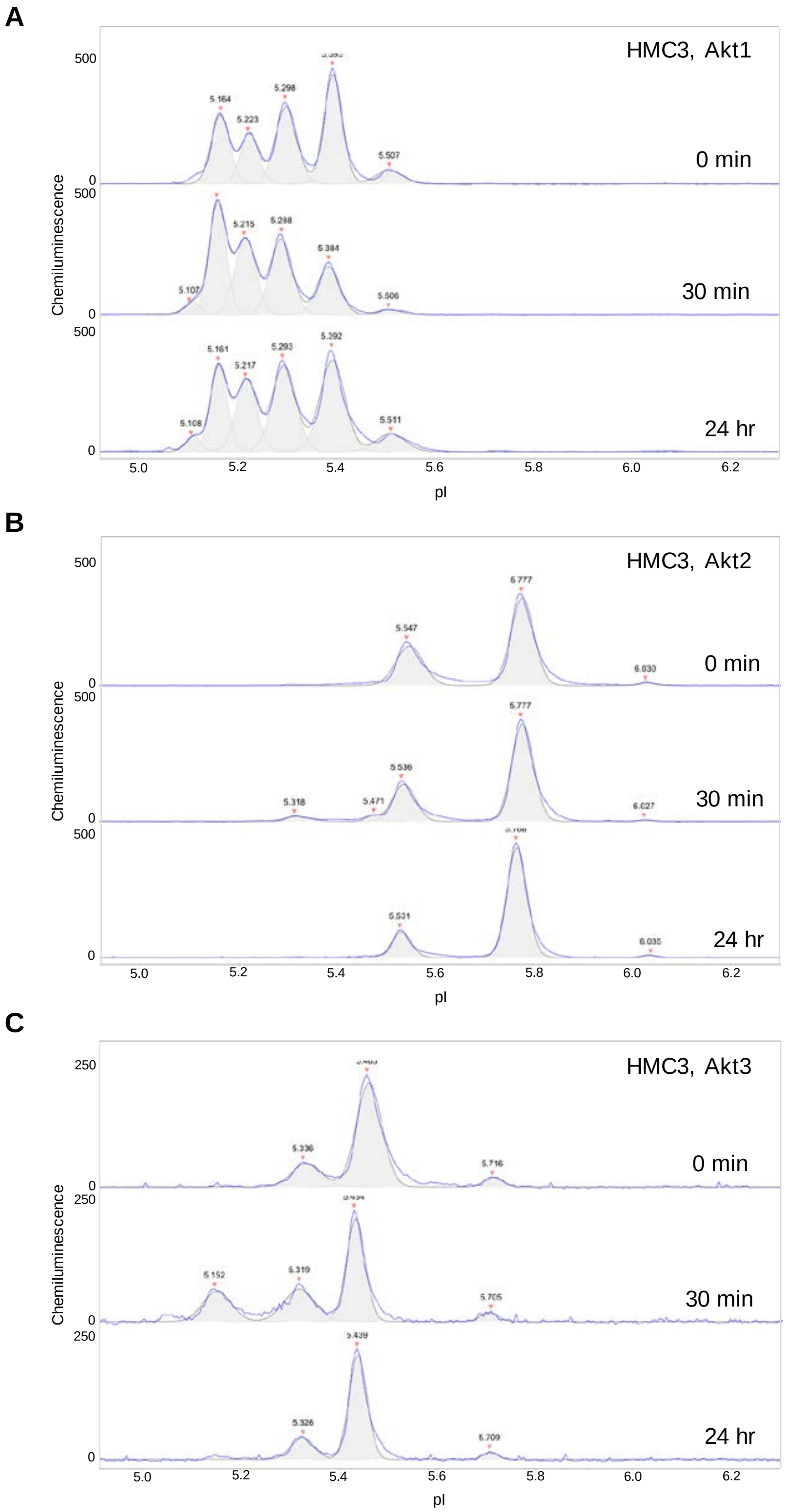

Supplementary Figure S2. Copaiba essential oil treatment transiently increases the phosphorylation of Akt1, Akt2, and Akt3 in HMC3 microglial cells. cIEF electropherograms of (A) Akt1, (B) Akt2, and (C) Akt3 as functions of time after treatment with copaiba essential oil.

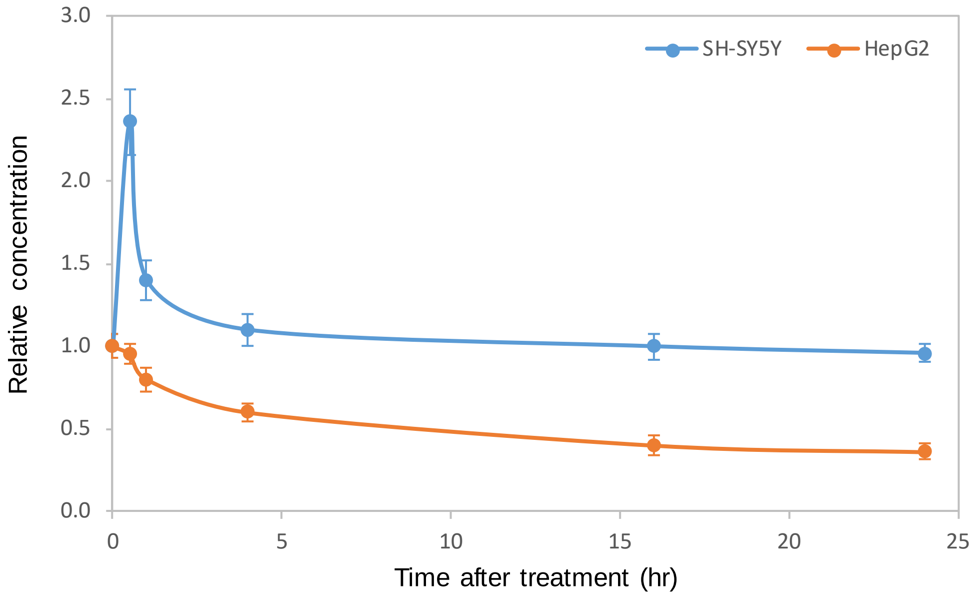

Supplementary Figure S3. Time-dependent regulation of Akt1 phosphorylation by copaiba essential oil in SH-SY5Y versus HepG2 cells. Relative concentration of Akt1 phosphoisoforms as a function of time after treatment with 100 ng/ml copaiba essential oil in SH-SY5Y (blue) versus HepG2 (orange) cells. The relative concentration describes the fold change in Akt1 phosphoisoforms after treatment compared to the control condition. The error bars are the standard deviations of six repeated measurements per experimental condition.

A

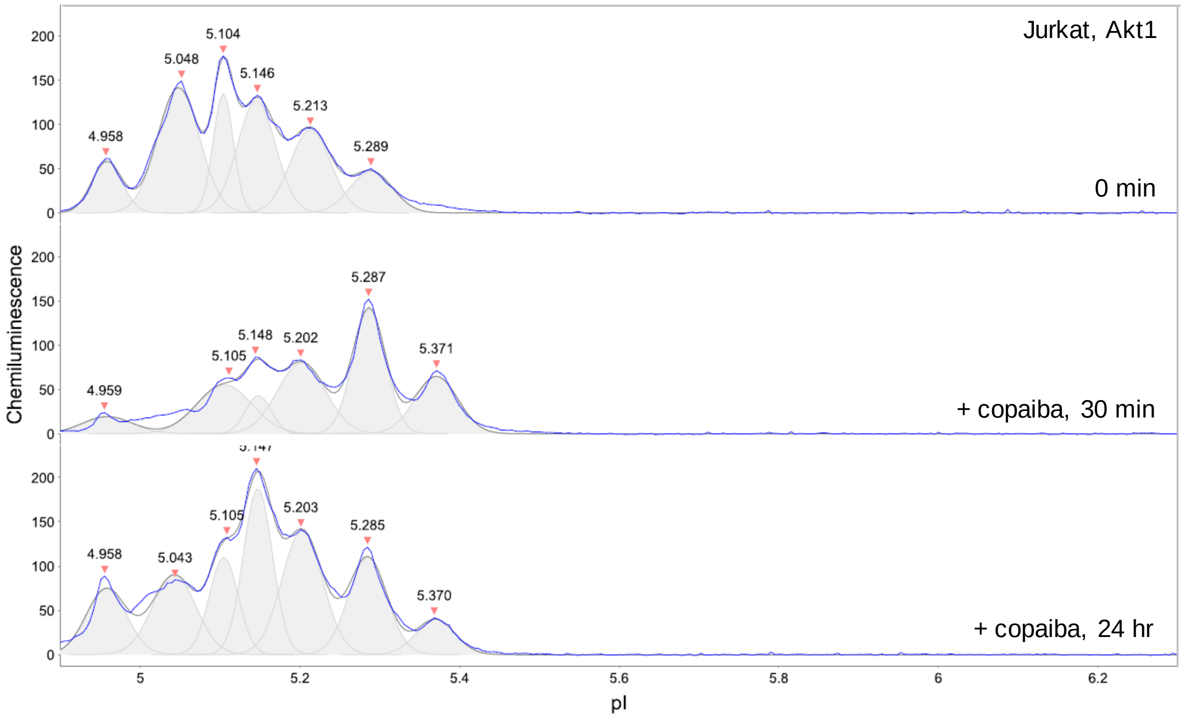

B

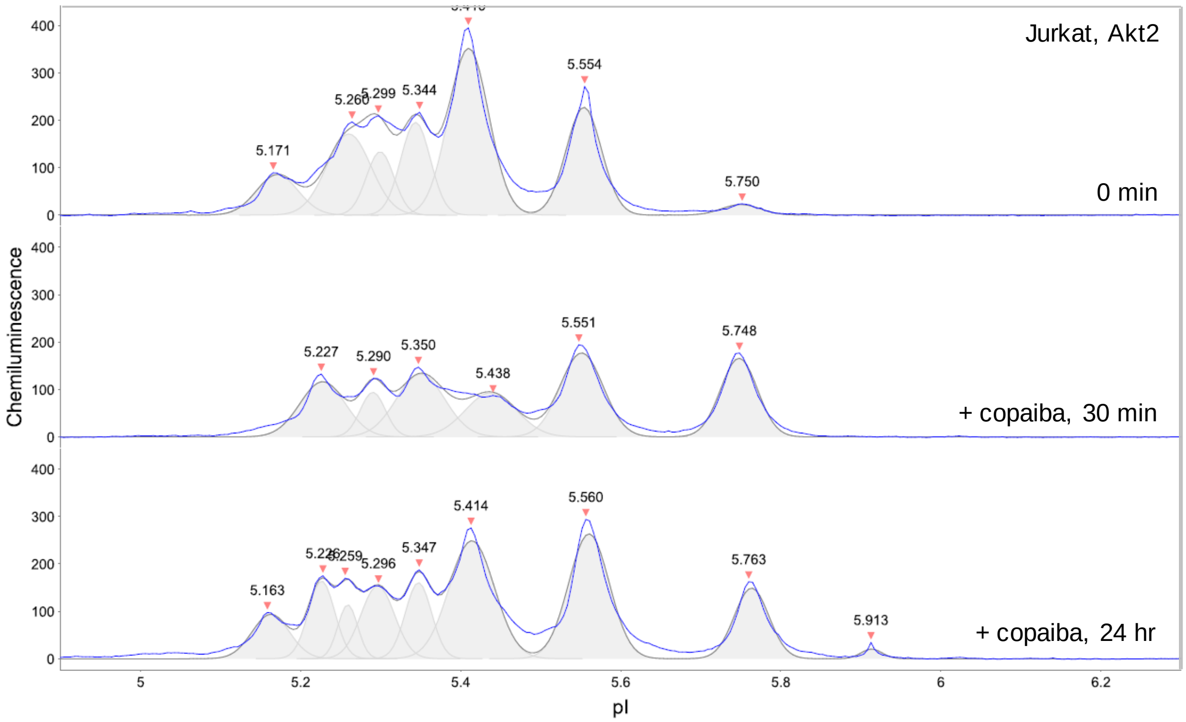

Supplementary Figure S4. Copaiba essential oil treatment reduces the phosphorylation of Akt1 and Akt2 in Jurkat T lymphocytes. cIEF electropherograms of (A) Akt1 and (B) Akt2 as functions of time after treatment with copaiba essential oil.

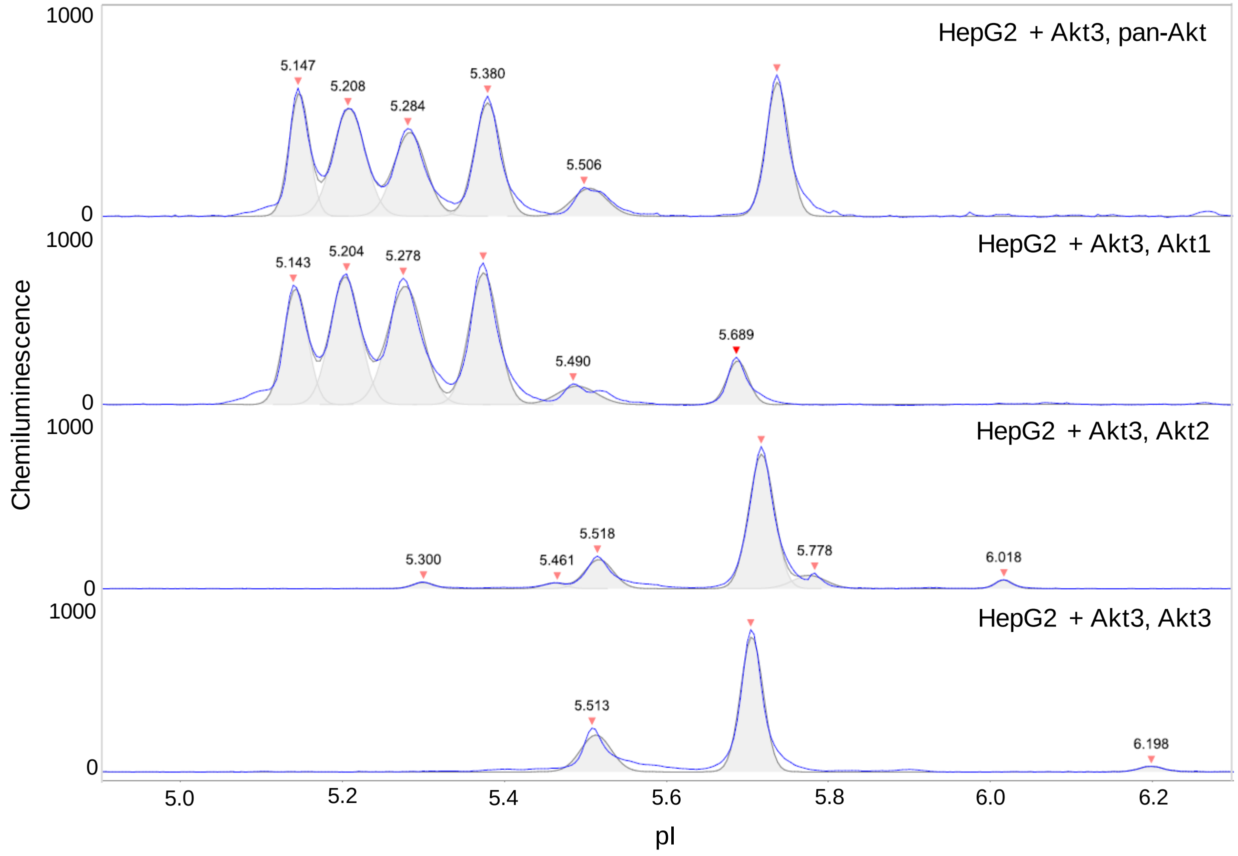

Supplementary Figure S5. Akt isoform identification in HepG2 cells transfected with Akt3-encoding plasmid DNA. cIEF electropherograms of pan-Akt (top panel), Akt1 (second panel), Akt2 (third panel), and Akt3 (fourth panel).

Supplementary Table S1. List of culture media

Cell Types	Culture Medium	Supplement	Atmosphere
SH-SY5Y	1:1 mixture of EMEM (cat. no. 302003, ATCC, Manassas, VA, USA) and F12K (cat. no. 302004, ATCC)	10% Fetal bovine serum (FBS, cat. no. SH30888.03, GE Life Sciences, Pittsburgh, PA, USA)	95% air, 5% CO_2
HepG2	RPMI1640 (cat. no. 11875, Gibco, Dublin, Ireland)	10% FBS & 1% MEM nonessential amino acids (cat. no. 25-025-CIR, Corning, NY, USA)	95% air, 5% CO_2
HMC3	EMEM	10% FBS	95% air, 5% CO_2
Jurkat	RPMI1640	10% FBS	95% air, 5% CO_2
Caco-2	EMEM	20% FBS	95% air, 5% CO_2
A549	F12K	10% FBS	95% air, 5% CO_2
HEK293	EMEM	10% FBS	95% air, 5% CO_2
MDA-MB-231	RPMI1640	10% FBS	95% air, 5% CO_2
Preadipocytes	PM1 (Zen-Bio, Research Triangle, NC, USA)	N/A	95% air, 5% CO_2
Adipocytes	AM1 (Zen-Bio)	N/A	95% air, 5% CO_2

Supplementary Table S2. List of primary and secondary antibodies

№	Antibody	Cat. No.	Vendor
1	pan-Akt	8312	Santa Cruz Biotech (Dallas, TX)
2	Akt1	2938	Cell Signaling (Danvers, MA)
3	Akt2	3063	Cell Signaling
4	Akt3	8018	Cell Signaling
5	mTOR	2983	Cell Signaling
6	p-mTOR (Ser2448)	5536	Cell Signaling
7	p70S6K	9202	Cell Signaling
8	p-p70S6K (Thr389)	9234	Cell Signaling
9	ERK1/2	040-474	Protein Simple (Santa Clara, CA)
10	STAT3	4904	Cell Signaling
11	β-actin	MAB8929	R&D Systems (Minneapolis, MN)
12	HSP60	F1800	R&D Systems
13	HSP70	4872	Cell Signaling
14	Secondary antibody (anti-rabbit HRP)	040-656	Protein Simple
15	Secondary antibody (anti-rabbit HRP)	042-206	Protein Simple
16	Secondary antibody (anti-mouse HRP)	042-205	Protein Simple
17	Secondary antibody (anti-rabbit NIR)	043-819	Protein Simple
18	Secondary antibody (anti-mouse NIR)	043-821	Protein Simple

Supplementary Table S3. List of biomarker proteins and their functions

№	Protein	Name	Function
1	Akt1	Protein kinase B, isoform 1	Apoptosis, proliferation, & cell migration
2	Akt2	Protein kinase B, isoform 2	Glucose metabolism
3	Akt3	Protein kinase B, isoform 3	Neuronal development
4	mTOR	Mechanistic target of rapamycin	Proliferation, motility, survival, autophagy,
5	p70S6K	Ribosomal S6 kinase β-1	Protein synthesis
6	ERK1/2	Mitogen-activated protein kinase 1/2	Proliferation, differentiation, development
7	STAT3	Signal transducer and activator of transcription 3	Immunity, proliferation, differentiation
